# Modeling preeclampsia using human induced pluripotent stem cells

**DOI:** 10.1038/s41598-021-85230-5

**Published:** 2021-03-15

**Authors:** Mariko Horii, Robert Morey, Tony Bui, Ojeni Touma, Katharine K. Nelson, Hee-Young Cho, Hannah Rishik, Louise C. Laurent, Mana M. Parast

**Affiliations:** 1 Department of Pathology, University of California San Diego, La Jolla, CA 92093 USA; 2 Sanford Consortium for Regenerative Medicine, University of California San Diego, La Jolla, CA 92093 USA; 3 Department of Obstetrics, Gynecology, and Reproductive Sciences, University of California San Diego, La Jolla, CA 92093 USA; 4 Department of Obstetrics and Gynecology, CHA Gangnam Medical Center, CHA University, Seoul, Korea

**Keywords:** Stem cells, Diseases

## Abstract

Preeclampsia (PE) is a pregnancy-specific hypertensive disorder, affecting up to 10% of pregnancies worldwide. The primary etiology is considered to be abnormal development and function of placental cells called trophoblasts. We previously developed a two-step protocol for differentiation of human pluripotent stem cells, first into cytotrophoblast (CTB) progenitor-like cells, and then into both syncytiotrophoblast (STB)- and extravillous trophoblast (EVT)-like cells, and showed that it can model both normal and abnormal trophoblast differentiation. We have now applied this protocol to induced pluripotent stem cells (iPSC) derived from placentas of pregnancies with or without PE. While there were no differences in CTB induction or EVT formation, PE-iPSC-derived trophoblast showed a defect in syncytialization, as well as a blunted response to hypoxia. RNAseq analysis showed defects in STB formation and response to hypoxia; however, DNA methylation changes were minimal, corresponding only to changes in response to hypoxia. Overall, PE-iPSC recapitulated multiple defects associated with placental dysfunction, including a lack of response to decreased oxygen tension. This emphasizes the importance of the maternal microenvironment in normal placentation, and highlights potential pathways that can be targeted for diagnosis or therapy, while absence of marked DNA methylation changes suggests that other regulatory mechanisms mediate these alterations.

## Introduction

Preeclampsia (PE), defined as new-onset hypertension associated with end-organ dysfunction (including thrombocytopenia, renal insufficiency, impaired liver function, pulmonary edema, or cerebral or visual disturbances) after 20 weeks’ gestation, is one of the most serious pregnancy complications, which can affect both mother and baby^1–3^. The incidence of PE has risen in the US, likely related to an increased prevalence of predisposing disorders, such as diabetes and obesity^3–5^. Specifically, early-onset (prior to 34 weeks gestation) PE with severe features is a major cause of maternal and fetal morbidity, including fetal growth restriction, and leads to adverse perinatal outcomes such as preterm delivery^2^. The pathophysiology of PE is still not completely clear, though abnormal differentiation and function of placental epithelial cells, called trophoblast, is thought to be a major component.

There are three main trophoblast cell types in the placenta; (1) cytotrophoblast (CTB), a self-renewing multipotent trophoblast progenitor cell; (2) syncytiotrophoblast (STB), which arise by cell–cell fusion of CTB in chorionic villi, and mediate nutrient/gas exchange and synthesize key pregnancy hormones; and (3) extravillous trophoblast (EVT), which arise from CTB that differentiate while migrating along the trophoblast cell column and invade the uterine wall, thereby anchoring the placenta to the uterus, while also remodeling maternal spiral arteries to establish the blood supply to the feto-placental unit^6,7^. The bulk of this uterine invasion and maternal vascular remodeling takes place prior to 10 weeks gestational age, while the placenta is developing under relative hypoxia^8^. In vitro culture of CTB under low oxygen has been shown to inhibit STB formation/function^9^, and enhance EVT differentiation through a hypoxia-inducible factor (HIF)-dependent mechanism^10^.

PE, particularly early-onset PE with severe features, is associated with specific findings on placental pathologic examination. These include a placental disc that is small (< 10th percentile for gestational age) and shows accelerated maturation (e.g. increased syncytial knots), central areas of infarction, perivillous fibrin deposition, and marginal/retroplacental hemorrhage (a sign of placental abruption)^11^. These lesions are secondary to underlying maternal vascular disease, which often manifests on placental exam as decidual vasculopathy. Decidual vasculopathy is characterized by lack of spiral arterioles remodeling by EVT, with vessels retaining their muscular wall and showing chronic inflammation and fibrinoid necrosis^11^. This constellation of findings is referred to as maternal vascular malperfusion (MVM) and has been associated with PE and/or fetal growth restriction, particularly in severe cases^12^. These histopathologic changes are accompanied by documented abnormalities in CTB, STB, and EVT^13^, as well as molecular evidence of oxidative stress as a result of abnormal maternal vascular remodeling^14–16^. The unique nature of these histologic and molecular findings accompanying the clinical disorder in humans has limited the utility of animal models of PE. Furthermore, to date, investigation of the underlying molecular mechanisms of this disease has been hampered by the lack of human trophoblast cells that accurately represent the early stages of placental development^17^.

Human pluripotent stem cells (hPSCs) were first reported to have trophoblast differentiation potential in 2002, when hCG-secreting multinucleated trophoblasts were identified in cultures containing feeder conditioned media (FCM) supplemented with bone morphogenetic protein-4 (BMP4)^18^. Since then, multiple groups, including ours, have used this model for the study of human trophoblast differentiation, using both embryonic stem cells (hESCs) and induced pluripotent stem cells (iPSC), and have identified signaling pathways and transcription factors that mediate this process^19–35^. Specifically, our group has identified that this differentiation can be modeled using a two-step protocol, by which pluripotent cells are first differentiated into p63^+^/CDX2^+^ CTB-like cells, and subsequently, into a mixture of terminally-differentiated hCG-secreting multinucleated STB-like cells and invasive HLA-G^+^ EVT-like cells^27,32^. We have shown that this protocol not only recapitulates normal trophoblast differentiation (including hypoxia-induced HIF-directed EVT differentiation), but can also be used to model placental disease (including the defect in STB differentiation seen in Trisomy 21-affected placentas). Most recently, we have applied knowledge about WNT-mediated mesoderm induction downstream of BMP4 signaling^31^ to further improve this protocol, adding the WNT inhibitor IWP2 to BMP4 in the first step, in order to restrict differentiation to the trophoblast lineage^36^.

Recently, Sheridan et al.^37^ used a similar protocol (using a combination of BMP4, A83–01, a TGF inhibitor, and PD173074, an FGF receptor inhibitor, or “BAP”) to explore the trophoblast phenotype of iPSCs from patients with early-onset PE with severe features and identified abnormalities in oxygen response mechanisms. However, they did not evaluate for defects in lineage-specific differentiation (into CTB, STB, or EVT) at the cellular level, and also limited their molecular evaluation to the transcriptome^37^. In this study, we used our updated two-step differentiation protocol to first explore the cellular phenotype seen in PE-iPSC derived trophoblast, and then expanded our analysis to evaluate changes in both gene expression and DNA methylation in order to identify the molecular mechanisms underlying this disease phenotype.

## Results

### Establishment of iPSC from umbilical cord-derived mesenchymal stem cells

Mesenchymal stem cells were derived from umbilical cord tissue (UC-MSC), as previously described^38,39^, from placentas of patients with and without early-onset (< 34 weeks gestation) preeclampsia (PE) with severe features, as defined by ACOG criteria^1^. To further select for cases with the most severe underlying trophoblast pathology, only cases that showed placental pathologic findings of maternal vascular malperfusion (MVM)^40^ were selected, including small placental disc (< 10th percentile for gestational age), and at least 2 of the following 5 lesions in the placental disc (hypermature, increase fibrin deposition, infarct, hematoma, decidual vasculopathy)^12^. Non-PE (control) cases were selected based on absence of clinical evidence of maternal hypertensive disease or fetal growth restriction, as well as absence of any features of MVM or other placental disc lesions. Table 1 shows demographic, clinical, and placental pathologic data from all cases.

**Table 1:** List of umbilical cord-derived MSCs used to generate iPSCs for this study, including patient demographic, clinical and placental pathology data.

Sample type	Cell line number	Maternal age at delivery	Ethnicity	Deliver mode	GA week (day)	Fetal sex	Clinical diagnosis	Birth weight percentile	Placental weight for GA	MVM	FVM
Non-PE	1938P	20	White	NSVD	26^1^	M	PTL	54	N	No	No
Non-PE	1947P	30	Hispanic	CS	34^0^	F	pPROM	27	N	No	No
Non-PE	1754P	37	White	CS	39^2^	M	Normal	51	N	No	No
PE	1932P	26	White	CS	32^4^	F	Severe PE/FGR	7	S	Yes	Yes
PE	1933P	33	Hispanic	CS	33^1^	M	Severe PE	27	S	Yes	No
PE	1981P	35	White	NSVD	33^2^	F	Severe PE/FGR	2	S	Yes	No

*NSVD* normal spontaneous vaginal delivery, *CS* cesarean section, *GA* gestational age, *M* male, *F* female, *PTL* preterm labor, *pPROM* preterm premature rupture of membranes, *PE* preeclampsia, *FGR* fetal growth restriction, *N* normal and *S* small for placental weight for GA, *MVM* maternal vascular malperfusion, *FVM* fetal vascular malperfusion.

Following reprogramming, 5 iPSC clones from each patient were taken through ten passages, and verified to be negative for Sendai virus integration by PCR; of these, at least 3 clones from each patient were confirmed to be pluripotent, based on the Pluritest assay^41^ (Supplementary Figure 1A), and to lack karyotypic abnormalities (Supplementary Figure 1B). We also profiled all iPSC lines (3 clones per patient line) using RNA sequencing (RNAseq), and confirmed that the undifferentiated iPSC clones from the same source pregnancy were as variable as those from different patients. The iPSC clones from the same patient did not cluster together using PCA (Supplementary Fig. 1C); also, correlation coefficients were similar between inter- and intra-patient iPSC clones (Supplementary Table 1). Therefore, it was deemed that factors other than genetic similarity were driving the differences between clones; thus, we treated each iPSC clone as a distinct cell line, with a total of nine PE and nine non-PE (control) iPSC lines, which we used for the following studies.

**Figure 1:**
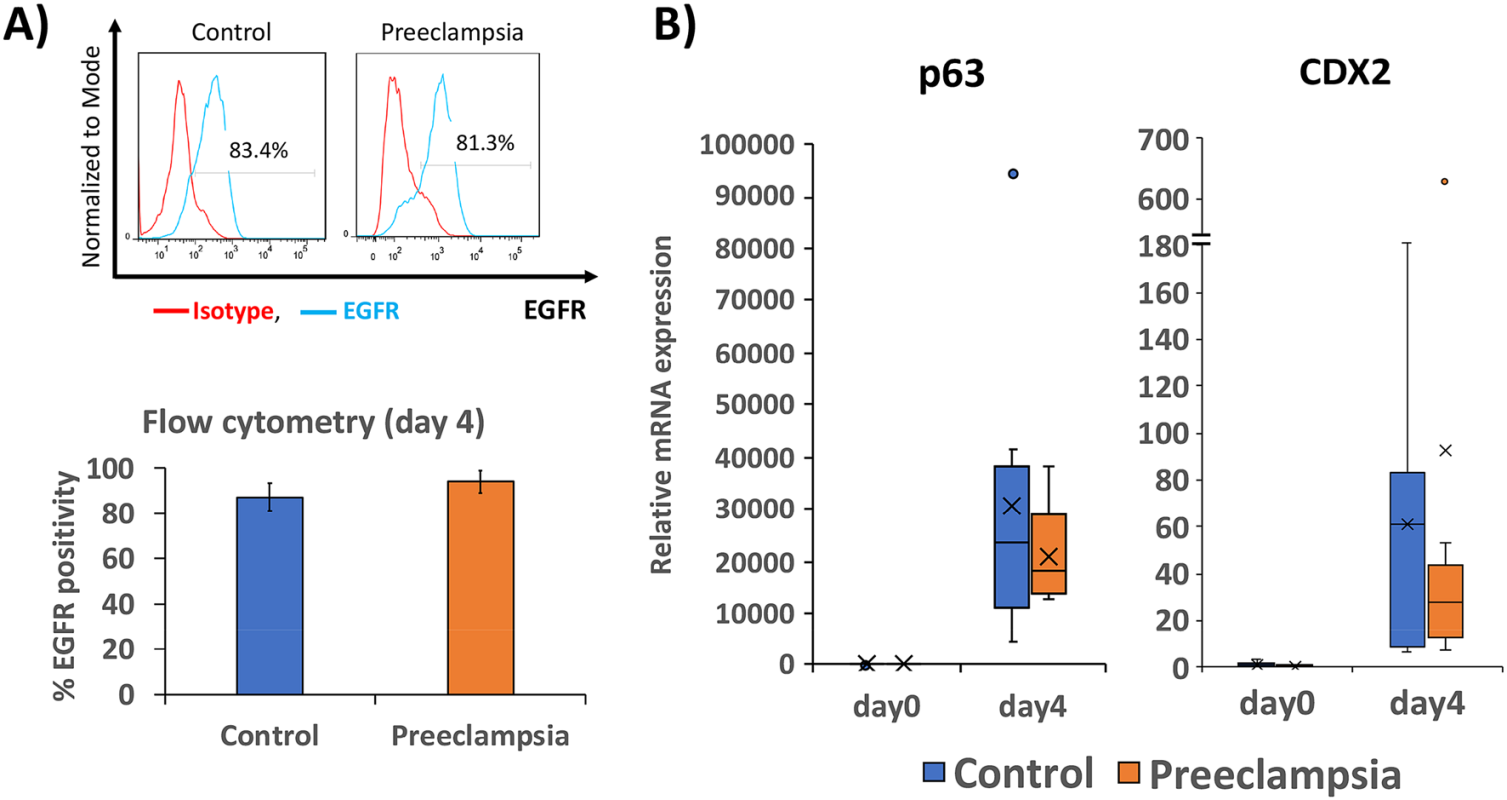
Differentiation of PE- and control-iPSC into CTB-like cells. (**A**) Upper panel: Representative flow cytometric analysis of CTB marker, EGFR, as compared to isotype control, following differentiation of iPSC into CTB-like cells (after 4 days of BMP4 + IWP2 treatment). Lower panel: Bar chart displaying average percent EGFR positive cells from both PE- and control-iPSC at day 4 of differentiation, ± standard deviation (n = 9 for each condition). (**B**) Box plot displaying qPCR of CTB markers p63 and CDX2 at day 4 of differentiation, normalized to L19, and expressed as fold change over undifferentiated control-iPSC (day 0) (n = 9 for each condition).

### CTB induction of PE and control iPSC

All 18 iPSC lines were subjected to our optimized two-step trophoblast differentiation protocol ^36^. Following culture in BMP4 and IWP2 (factors used to induce CTB), cells displayed changes in morphology starting on day 1. By day 4, all cells had flattened to produce a uniform epithelial morphology. Cells were assessed for surface expression of EGFR, a CTB marker, by flow cytometry, and over 80% of both PE and control iPSC-derived cells expressed EGFR (Fig. 1A). In addition, quantitative RT-PCR (qPCR) showed similar transcript levels for CDX2 and p63, markers of early gestation CTB, between PE and control iPSC-derived cells (Fig. 1B). Based on the above, we concluded that CTB induction is not compromised in PE, compared to control, iPSCs.

### Terminal trophoblast differentiation of PE and control iPSC

We next subjected all 18 iPSC-derived CTB to the second step of differentiation, by treating the cells with FCM + BMP4 for an additional 4 days (day + 4), under either 21% oxygen, which is known to promote differentiation into STB, or 2% oxygen, which promotes differentiation into EVT^32,36^.

**Figure 2:**
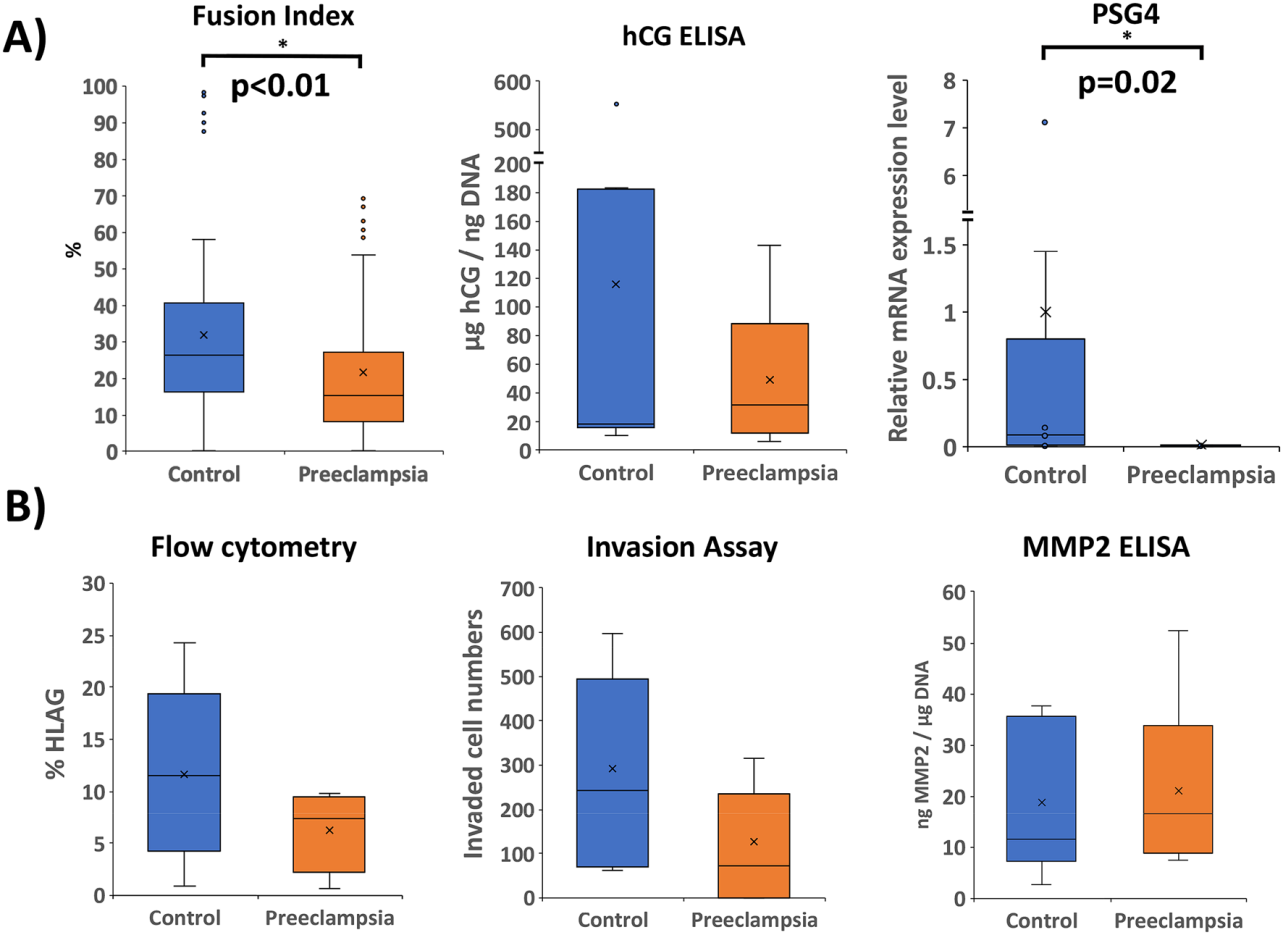
Differentiation of iPSCs into either STB-like cells (performed under 21% oxygen) (**A**) or into EVT-like cells (performed under 2% oxygen) (**B**). STB formation and function (**A**) was assessed by calculation of fusion index, hCG secretion (normalized to DNA content), and PSG4 expression level (by qPCR, normalized to L19, and shown as fold change compared to control-iPSC in the same differentiation state). EVT formation and function (**B**) was assessed by expression of surface HLA-G as measured by flow cytometry, invasive capacity as assessed by Matrigel invasion assay, and MMP2 secretion (normalized to DNA content). *Indicates statistical significance by Mann–Whitney U, with *p* values as stated (n = 9 for each condition).

Differentiation into STB (under 21% oxygen) was quantified based on morphology (using fusion index calculation), secretory function (hCG ELISA), and marker expression (by qPCR). We found that PE-iPSC-derived trophoblast had a reduced fusion index (21.6% in PE vs. 31.8% in control; *p* < 0.01) (Fig. 2A); this was not due to the differences in total number of nuclei (Supplementary Figure 2A), nor in the total number of cells (Supplementary Fig. 2B) between control and PE iPSC-derived STB. Rather, it appears that PE-iPSC lines have fewer cells/nuclei that fuse into STB (Supplementary Figure 2C–E). While we defined STB, as we have in the past^32^, as cells containing at least 3 nuclei, to further evaluate size of STB patches between PE- and control-iPSC-derived trophoblast, we also compared STB with 3–10 nuclei, 11–30 nuclei, or 31 or more nuclei (Supplementary Fig. 2F). Compared to control, PE-iPSCs show more non-fused cells (cells with 1–2 nuclei) in the overall area, with a higher number of fused cells with 11–30 nuclei or 31 or higher nuclei; however, these differences were not statistically significant. In addition, the corresponding median cell numbers were also not significantly different. Therefore, overall, the main difference was in the fusion efficiency, which was lower in PE-iPSC-derived trophoblast, as best reflected by the fusion index. PE-iPSC-derived trophoblast did not show a significant reduction in hCG secretion, and, by qPCR, only PSG4 was significantly down-regulated in PE-STB (183-fold; *p* = 0.02 (Fig. 2A), without alteration of other STB-associated genes (GCM1, ERVW-1, and CGB) (data not shown).

We next evaluated EVT differentiation and function (under 2% oxygen), again using a combination of flow cytometry (for the EVT surface marker HLA-G), Matrigel invasion assay, secretory function (MMP2 ELISA), and marker expression (by qPCR). We found no significant differences in HLA-G by flow cytometry, nor in invasion or MMP2 secretion, between the PE and control iPSC-derived trophoblast (Fig. 2B); also, by qPCR, there were no differences in expression of HLA-G (data not shown). These data suggest that under our STB and EVT differentiation conditions, only STB formation and maturation, but not STB secretory function, nor EVT formation or function, were affected in PE-iPSC.

### Abnormal responses to changes in oxygen tension

**Figure 3:**
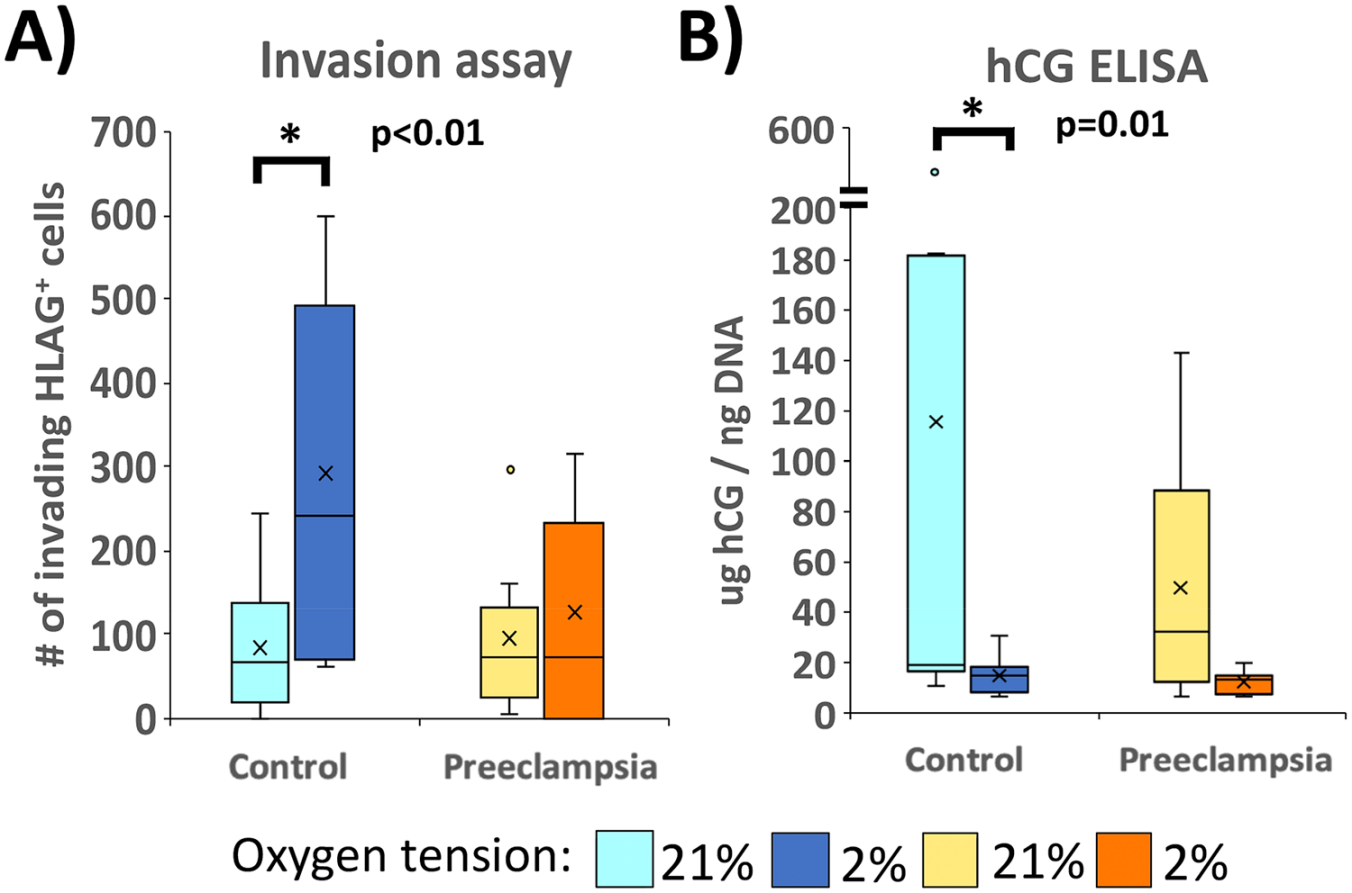
Response of iPSC-derived trophoblast to changes in oxygen tension. (**A**) Invasive capacity was assessed by Matrigel invasion assay and (**B**) hCG secretion was measured by hCG ELISA and normalized to its DNA content. Only control iPSC-derived trophoblast (but not PE iPSC-derived trophoblast) show a statistically significant response to a lowering of oxygen tension. *Indicates statistical significance by Wilcoxon Signed Rank Test, with *p* values as stated (n = 9 for each condition).

We next assessed the responses of iPSC-derived trophoblast to differing oxygen tensions, comparing both hCG secretion and Matrigel invasion of PE and control lines at the conclusion of step 2 of differentiation (day + 4), conducted under either 21% or 2% oxygen. As expected, compared to the phenotype under 21% oxygen, control iPSC-derived trophoblast showed a 3.8-fold increase in number of invasive HLA-G^+^ cells (*p* < 0.01, Fig. 3A), as well as an 8.2-fold reduction in hCG secretion (*p* = 0.01, Fig. 3B) under 2% oxygen. However, PE-iPSC-derived trophoblast had blunted responses to this difference in oxygen tension, lacking significant differences in invasion or hCG secretion between the different oxygen tensions (Fig. 3A,B). These data indicate that, while EVT formation and STB secretory function are not categorically abnormal in PE, the formation and/or function of these cells in response to changes in the microenvironment are what underly the pathophysiology of this pregnancy disorder.

### Global gene expression in PE and control iPSC-derived trophoblast

**Figure 4:**
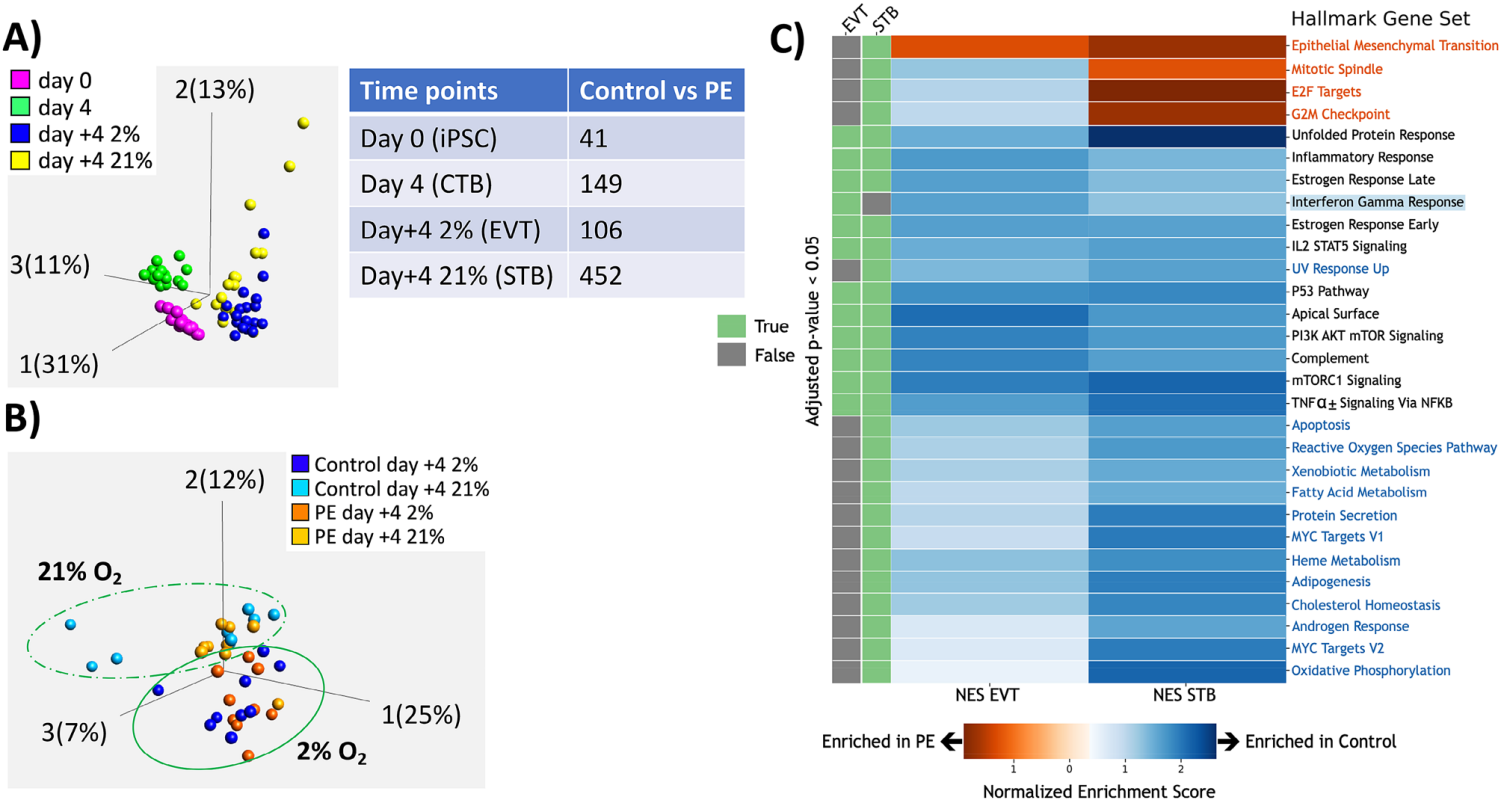
Analysis of RNAseq data with respect to differentiation state. Data from iPSC at day 0 (iPSC), and iPSC-derived trophoblast at day 4 (cytotrophoblast/CTB-like), day + 4 at 2% oxygen (extravillous trophoblast/EVT-like) and day + 4 at 21% oxygen (syncytiotrophoblast/STB-like). (**A**) PCA plot (left) showing iPSC at different timepoints of trophoblast differentiation, and table (right) showing the number of differentially expressed genes (DEGs) between control- and PE-iPSC at each of these time points. (**B**) PCA plot displaying only the day + 4 timepoints, with dashed line indicating greater difference in gene expression between control- and PE-iPSC differentiated into STB-like cells (under 21% oxygen) and solid line showing closer clustering of control- and PE-iPSC differentiated into EVT-like cells (under 2% oxygen). (**C**) Gene Set Enrichment Analysis (GSEA) of iPSC-derived trophoblasts at day + 4. Heatmap displays normalized enrichment score (NES), with orange color showing gene-sets enriched in PE, and blue color showing gene-sets enriched in control. Green bars (left) indicate those gene-sets with *p* value < 0.05 in either 2% (EVT-like state) or 21% (STB-like state) oxygen tension. Gene-sets written in orange and blue font are uniquely enriched in PE- or control-iPSC-derived trophoblast, respectively, under 21% oxygen (STB-like state). Only one pathway (highlighted in blue) was uniquely enriched in control-iPSC-derived trophoblast under 2% oxygen (EVT-like state).

To further probe the etiology for these phenotypic differences between PE and control iPSC-derived trophoblast, we evaluated global gene expression of all iPSC lines at day 0 (undifferentiated), day 4 (CTB-state), day + 4 under 21% oxygen (STB-state), and day + 4 under 2% oxygen (EVT-state) by RNAseq. Principle component analysis (PCA) showed that the greatest amount of difference between these samples was temporal (PC1: 31%) (Fig. 4A, left), followed by variation between the STB- and EVT- differentiation states (PC2: 13%), and variation between day 4 and other time points (PC3: 11%). Separation between disease phenotype accounted for only 2% of the overall variance in gene expression (PC6). We next performed differential expression analysis within each differentiation timepoint, and noted that the number of differentially expressed genes (DEG) was highest between PE and control iPSC-derived STB (Fig. 4A, right, Supplementary Table 4), correlating with the noted differences in STB differentiation phenotype seen at day + 4 under 21% oxygen (see Fig. 2).

To further investigate gene expression differences in terminally differentiated trophoblast derived from our iPSC lines, we performed PCA with control and PE iPSC lines at day + 4 of differentiation, under both 21% and 2% oxygen. We found that the greatest variance among these samples was due to control-iPSC at day + 4 in 21% oxygen, followed by variance between samples at different oxygen tensions (Fig. 4B). Specifically, there was greater difference between control and PE-iPSC at 21% oxygen (STB-like cells, light blue vs. light orange circles in Fig. 4B), compared to control and PE-iPSC at 2% oxygen (EVT-like cells, dark blue vs. dark orange circles in Fig. 4B), recapitulating the number of DEGs between control and PE in these two different conditions (Fig. 4A, right, Supplementary Table 4).

We next performed Gene Set Enrichment Analysis (GSEA) focusing on differences between PE- and control-iPSC at day + 4 of differentiation in order to evaluate differences between control- and PE-iPSC-STB or iPSC-EVT. Using MSigDB Hallmark gene sets^42^, a total of 28 gene sets were significantly enriched (adjusted *p* value < 0.05) in the comparison between the PE and control STB-like cells (at day + 4 in 21% oxygen) (“STB gene sets,” Supplementary Table 2), while only 12 gene sets were significantly enriched (adjusted *p* value < 0.05) in the comparison between the PE and control EVT-like cells (at day + 4 in 2% oxygen) (“EVT gene sets,” Supplementary Table 3). Of the 28 STB gene sets, 4 were uniquely enriched in PE-iPSC-STB (orange font, Fig. 4C), and 13 were uniquely enriched in control-iPSC-STB (blue font, Fig. 4C). Conversely, of the 12 EVT gene sets, none were uniquely enriched in PE-iPSC-EVT, and only one, “interferon gamma response,” was uniquely enriched in control-iPSC-EVT (blue highlight, Fig. 4C); the latter gene set contained only one statistically significant leading edge gene (see Supplementary Table 3).

Since control iPSC had a stronger STB phenotype (see Fig. 2 above), we focused first on the 13 gene sets uniquely enriched in these cells (blue font, Fig. 4C). Several of the gene sets are involved in cellular metabolism (including adipogenesis, cholesterol homeostasis, heme metabolism, and protein secretion), and several others in cellular stress response (apoptosis, UV response, and reactive oxygen species). Of the leading edge genes within these gene sets (see Supplementary Table 2), several stand out based on their known functions. ADM (adrenomedullin), whose expression/secretion is known to be reduced in STB from PE placentae^43^, was enriched 2.4-fold in control- over PE-iPSC derived STB. GPX3 and GPX4, members of the glutathione peroxidase family known to play crucial roles in defense against oxidative stress^16,44,45^, showed 3.6-fold and 1.5-fold higher expression, respectively, in STB derived from control-iPSC vs. PE-iPSC. Activating Transcription Factor 3 (ATF3), associated with both apoptosis and UV response gene sets, was enriched threefold in control-iPSC-derived STB. ATF3 is also involved in protection against cellular stress responses, including oxidative stress and DNA damage^46,47^. Reticulon-3 (RTN3), which was enriched 1.6-fold in STB derived from control-iPSC, is involved in clearance of protein aggregates and ER stress and is a known negative regulator of amyloid-beta production^48^. These data suggest similarities between primary PE-associated and PE-iPSC-derived STB, and suggest that STB derived from PE-iPSC may be more susceptible to environmental stressors.

Within the 4 gene sets significantly enriched in PE-iPSC-STB, we noted epithelial-mesenchymal transition (EMT) (Fig. 4C); this gene set included several prominent leading edge genes, including decorin (DCN), and periostin (POSTN), which were enriched sixfold, and 3.8-fold respectively, in PE-iPSC-STB. DCN a member of the small leucine-rich proteoglycan family of proteins^49^, and POSTN an extracellular matrix protein^50^; expression of these genes is increased, either in placental tissues or serum from patients with preeclampsia^49,50^. The other three gene sets enriched in PE-iPSC-STB were all involved in cell division, and included mitotic spindle, G2M checkpoint, and E2F target gene sets.

**Figure 5:**
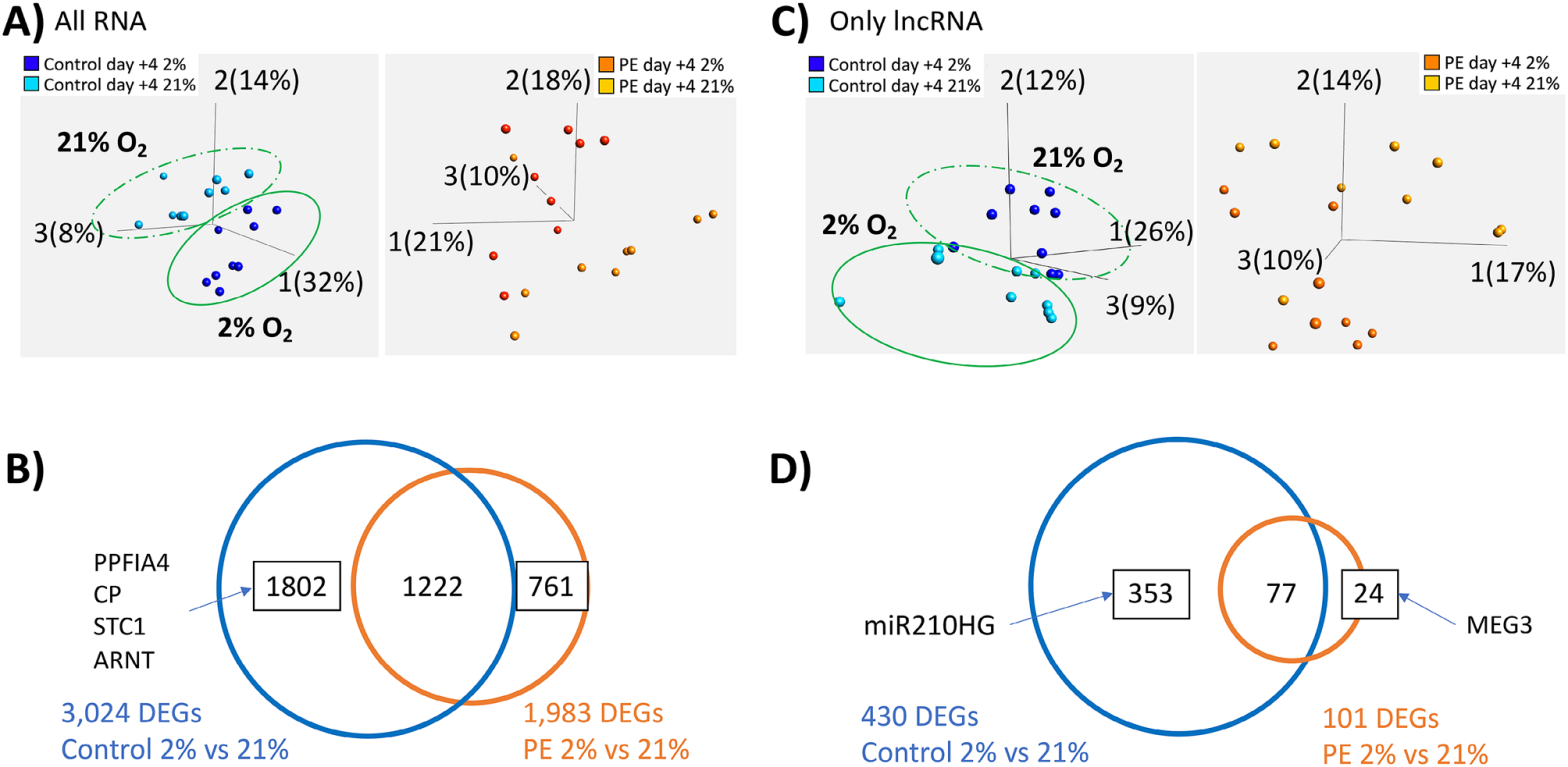
Analysis of RNAseq data with respect to response to oxygen tension. (**A**) PCA plot displaying either control-iPSC-derived trophoblast (left), or PE-iPSC-derived trophoblast (right). Trophoblast derived from control-iPSC separate much more clearly based on oxygen tension, compared to those derived from PE-iPSC. (**B**) Venn diagram displaying the number of DEGs between different oxygen tensions (2% vs. 21%) within the same disease condition (control and PE). Boxed numbers indicate DEGs unique to either iPSC group, with gene symbols indicating a handful of these unique genes in trophoblast derived from control-iPSC. (**C**) PCA plot displaying only long non-coding RNAs (lncRNAs) in either control-iPSC-derived trophoblast (left), or PE-iPSC-derived trophoblast (right). Based on analysis of this RNA subset, trophoblast derived from control-iPSC again separate much more clearly based on oxygen tension, compared to those derived from PE-iPSC. (**D**) Venn diagram displaying the number of differentially expressed lncRNAs between different oxygen tensions (2% vs. 21%) within the same disease condition (control and PE). Boxed numbers indicate number of differentially expressed lncRNAs unique to either iPSC group, with gene symbols indicating unique lncRNAs in either group.

Finally, we evaluated our RNAseq data in the context of the phenotypic difference in response to hypoxia seen in our iPSC model (see Fig. 3). PCA of control- and PE-iPSC at day + 4 showed more distinct clustering by oxygen tension in the control-iPSC (Fig. 5A, left), than in PE-iPSC (Fig. 5A, right). In order to identify genes responsible for this phenotype, we performed differential expression analysis between control-iPSC-derived CTB differentiated in 21% vs. 2% oxygen, and PE-iPSC-derived CTB differentiated in this way. There were 3,024 DEGs between the control-iPSC differentiated under these two oxygen tensions, compared to only 1,983 DEGs from PE-iPSC (Fig. 5B, Supplementary Table 5). After removing the overlapping genes, we identified 1,802 and 761 DEGs specific to oxygen response of control- and PE-iPSC-derived trophoblast, respectively (Fig. 5B, Supplementary Table 5). Within those DEGs unique to control-iPSC, we found PPFIA4 (also known as Liprin-α4), ceruloplasmin (CP), and stanniocalcin-1 (STC1), which were increased 13.6-fold, 77-fold, and fivefold, respectively, under 2% oxygen, only in control iPSC-derived trophoblast (Supplementary Figure 4). Both liprin-α4 and STC1 have been shown to promote invasion of cancer cells^51–53^, while secretion of ceruloplasmin in the placenta is known to be induced by hypoxia^54^. Another interesting gene in this group was ARNT (aryl hydrocarbon receptor nuclear translocator; also called “HIF1β”), which showed a statistically-significant (adjusted *p* value < 0.05), albeit modest (1.3-fold), increase only in control-iPSC (Supplementary Figure 4). In combination with one of several “HIF1α” subunits, ARNT forms the transcriptionally active hypoxia-inducible complex, which regulates the primary response of cells to a decrease in oxygen tension^55^.

There is growing evidence that long non-coding RNAs (lncRNAs), a class of non-protein coding RNA molecules with a length of over 200 nucleotides, are involved in regulation of gene expression in response to hypoxia, at the genomic, transcriptional, and post-transcriptional levels^56^. To ascertain if phenotypic differences in response to hypoxia could be partly attributed to differences in lncRNAs, we also performed differential gene expression on all Gencode (v.35) long noncoding RNAs extracted from our rRNA depleted RNAseq data. We performed this subset analysis to better detect differentially expressed lncRNAs, which are often expressed at a lower level compared to protein-coding RNAs^57^. Similar to the PCA analysis of all genes at day + 4, the lncRNA subset PCA showed more distinct clustering by oxygen tension in the control-iPSC (Fig. 5C, left) than in PE-iPSC (Fig. 5C, right). Moreover, differential expression analysis showed that there was a substantially higher number of differentially expressed lncRNAs (adjusted *p* value < 0.05) between control iPSC-derived CTB differentiated in 21% vs 2% oxygen, compared to PE iPSC-derived CTB differentiated in the same manner (Fig. 5D, Supplementary Table 6). After removing the overlapping lncRNAs, there were only 24 uniquely differentially expressed lncRNAs in the PE-iPSC-derived trophoblast, but over 350 lncRNAs in the control-iPSC-derived trophoblast (Fig. 5D, Supplementary Table 6). The latter included miR-210HG (miR-210 host gene**,** Supplementary Figure 4); this is a precursor form of miR-210, which is known as a downstream effector of HIF action^58^ and previously shown to be dysregulated in PE^59^. Conversely, among the lncRNAs uniquely downregulated in PE-iPSC-derived trophoblast under 2% oxygen was MEG3 (Supplementary Figure 4). MEG3 promotes HIF-1α protein translation^60^, and is down-regulated in placentas from PE pregnancies^61^.

All together, these data confirm that control-iPSC-derived trophoblast show significantly greater gene expression changes in response to hypoxia, and that these changes in gene expression are consistent with the cellular effects on invasive and secretory phenotypes uniquely noted in the control-iPSC-derived trophoblast, and blunted/lacking in the PE-iPSC-derived trophoblast.

### Assessment of the epigenome in MSCs and iPSCs from PE and non-PE control placentas

**Figure 6:**
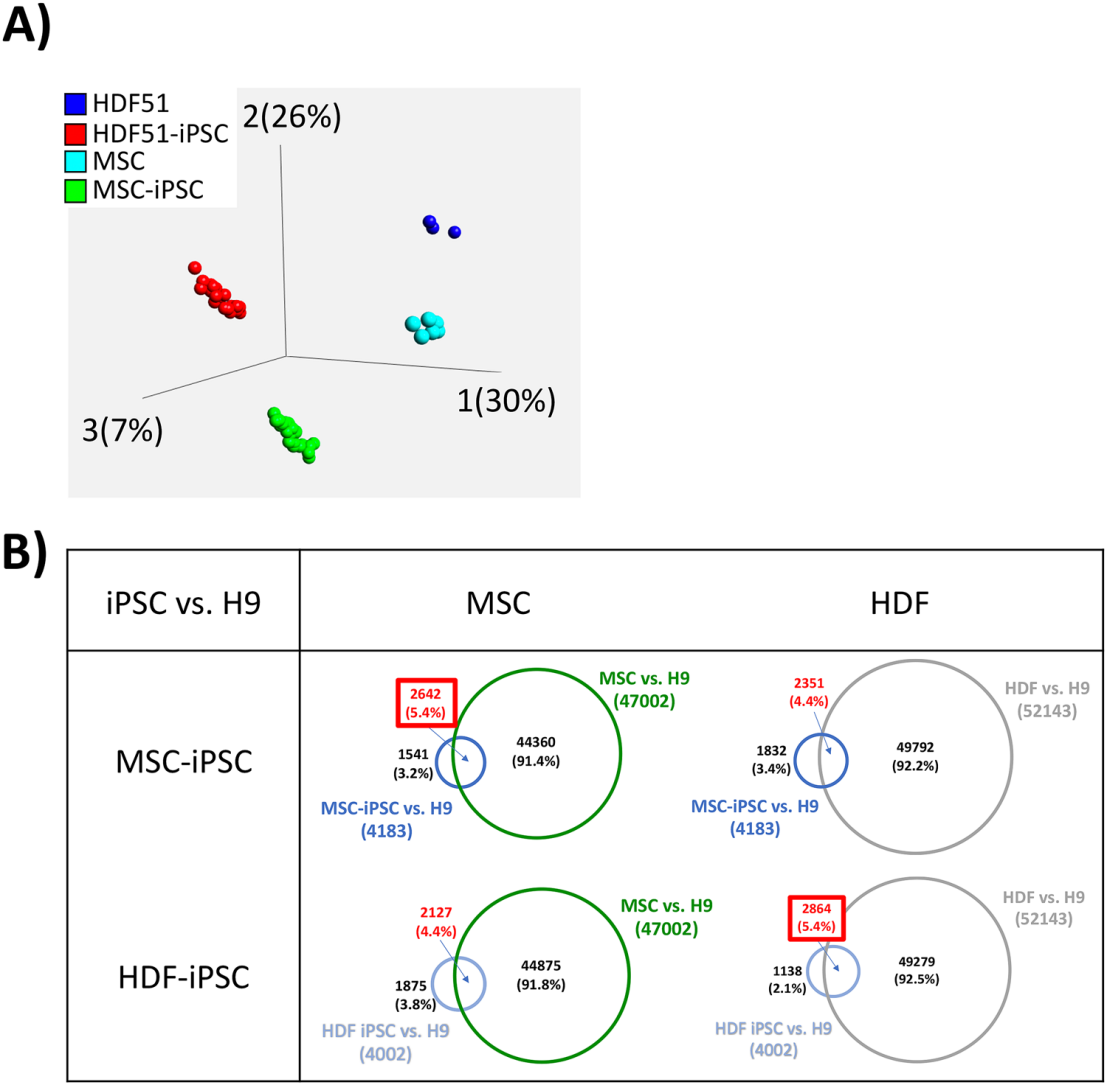
DNA methylation analysis, comparing iPSCs to their cells-of-origin. (**A**) PCA plot displaying methylation data from our 6 mesenchymal stem cells (MSC) and 18 MSC-derived iPSC (3 iPSC clones per MSC), and previously-obtained methylation data from human dermal fibroblasts (HDF) and HDF-derived iPSCs. (**B**) Table displaying the number of differentially expressed probes (DMP) shared with H9/WA09 human embryonic stem cell line, using Venn diagrams. Overlapping probes of each combination were compared, with each cell type having a higher number of overlapping DMPs when compared to iPSC of the same cell-of-origin. DMPs were determined using a Δβ-value of 0.3 or above; cross reacting and sex chromosome probes were excluded from this analysis.

Previous reports indicate that iPSCs retain both cell-of-origin-specific^62,63^ and donor-specific epigenetic marks^64,65^. To determine whether our MSC-derived iPSCs retain such epigenetic marks, we first compared their global DNA methylation to source MSCs, human dermal fibroblasts (HDF), and matched HDF-derived iPSCs using EPIC/450 k DNA methylation arrays. Principle component analysis (PCA) showed that each cell type clustered tightly based on their DNA methylation patterns, with the greatest differences seen between primary cells and iPSCs (PC1, 30%), followed by differences between MSCs and HDFs (PC2, 26%) (Fig. 6A). We further compared the number of differentially methylated probes (DMPs) in these iPSCs to the embryonic stem cell line, WA09/H9. We noted that MSC- and HDF-derived iPSCs have more probes in common with their respective cells-of-origin, primary MSCs and HDFs, respectively (Fig. 6B). These data suggest that, based on DNA methylation data, MSC-iPSCs retain some cell-of-origin epigenetic memory.

**Figure 7:**
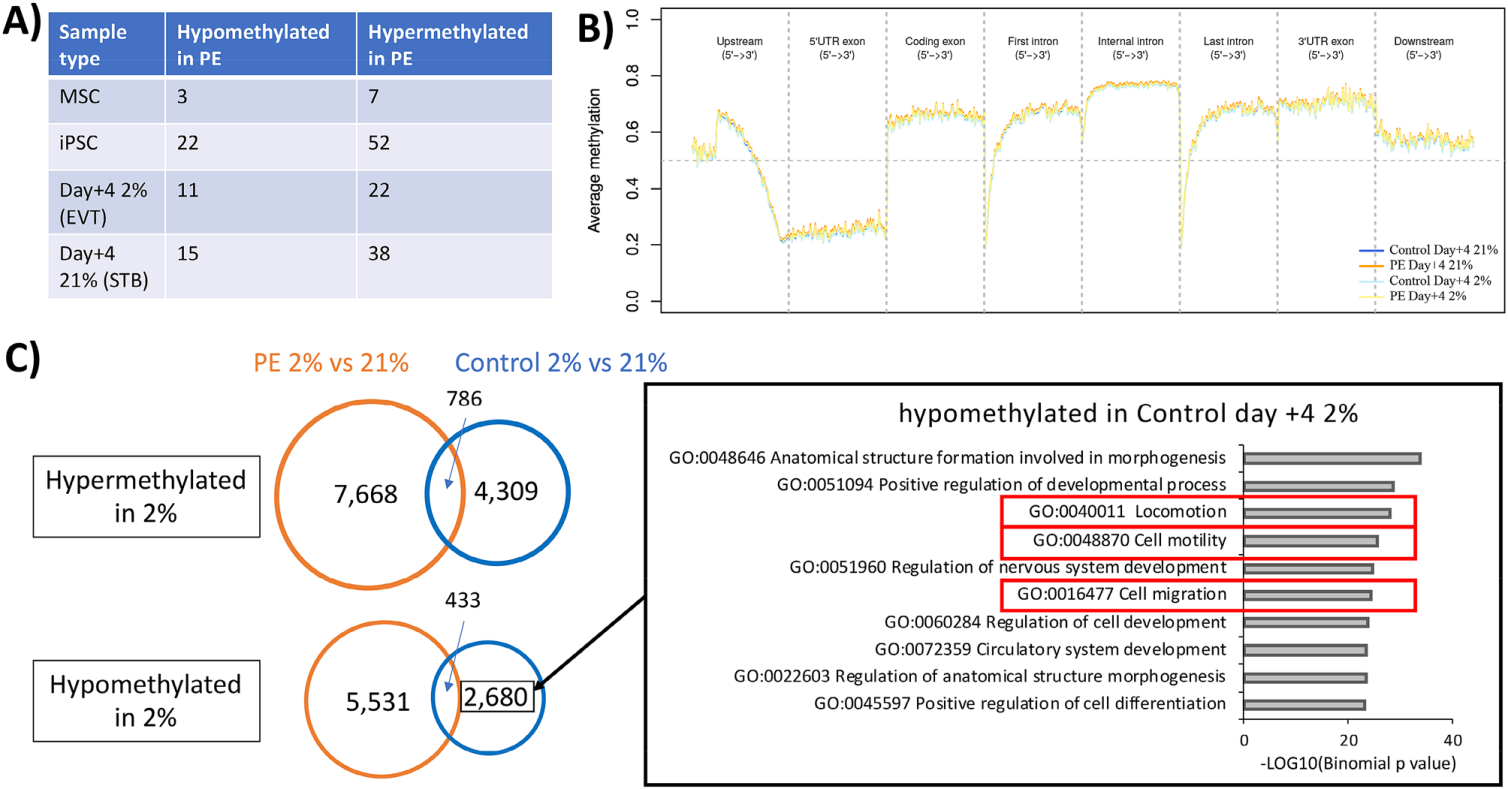
DNA methylation analysis with respect to iPSC phenotype. (**A**) Table displaying the number of DMPs between PE and control MSC, undifferentiated iPSC, and iPSC at day + 4 of differentiation (at either 2% or 21% oxygen) (n = 6 for each comparison). DMPs were determined using a Δβ-value of 0.3 or above; cross reacting and sex chromosome probes were excluded from this analysis. (**B**) Average β-value plot showing PE and control at day + 4 differentiation, with genomic regions around coding genes. (**C**) Venn diagram showing the number of DMPs (based on trichotomized data), hyper- or hypo- methylated in either control- or PE-iPSC-derived trophoblast, with summary of GREAT output and top 10 GO terms from DMPs uniquely hypomethylated in control-iPSC derived trophoblast under 2% oxygen, displayed in the right panel.

We next asked whether PE-specific DMPs were retained from the parental MSCs, through reprogramming to iPSC and differentiation to trophoblast. DMP analysis showed very few probes were differentially methylated (FDR < 0.05) when comparing the PE- and control-iPSC derived trophoblast at each time point (Fig. 7A). Among the 850 K probes, there were only three to five probes each that remained significantly differentially methylated in PE through differentiation, when compared to control iPSC. Overall, there were more hypermethylated regions in PE-iPSC at each step of differentiation. However, when we evaluated these DMPs, we were unable to find a link between the noted phenotypes in PE-iPSC-derived trophoblast and this small number of DMPs (data not shown).

We next evaluated the global DNA methylation profile (median β-value) of our PE- and control-iPSC-derived trophoblast at day + 4 (at both oxygen tensions), for genomic regions around genes, in order to correlate changes in methylation to changes in gene expression. We first evaluated all probes (Fig. 7B), then focused on probes associated with 452 DEGs in PE- vs. control-iPSC-derived STB (day + 4 under 21% oxygen) (see Fig. 4A, right; Supplementary Figure 6A), and subsequently on probes associated with 1802 DEGs unique to control-iPSC-derived trophoblast’s response to hypoxia (see Fig. 5B; Supplementary Figure 6B). We noted that DNA methylation profiles of differentiated iPSCs were very similar (Fig. 7B, Supplementary Figure 6A-B), which was consistent with our earlier DMP analysis, which required DMPs to have a Δβ-value greater than 0.3, resulting in very few probes to identified as differentially methylated (see Fig. 7A).

To enable detection of small changes in methylation, we trichotomized the β-value into three categories; un-methylated, semi-methylated, and full-methylated; transforming the β-value into 0, 1, 2, respectively^66^. Using these trichotomized data, we identified a larger number of DMPs, with the number of probes that consistently remained in the DMPs through reprogramming and differentiation ranging from ~ 560–3000 (568 and 564 hypomethylated DMPs maintained through differentiation at day + 4 in 21% and 2% oxygen, respectively; and 2927 and 2614 hypermethylated DMPs maintained through differentiation at day + 4 in 21% and 2% oxygen, respectively). In order to evaluate the association between these DMPs and the differentiation defect associated with PE-iPSC, we performed functional analysis using the Genomic Regions Enrichment of Annotations Tool (GREAT)^67^ with data from both control- and PE-iPSC at day + 4 of differentiation under 21% oxygen; however, we did not identify any functional terms that explained the STB defect noted in the PE-iPSC.

We next evaluated the trichotomized probe data with respect to response to hypoxia, focusing on probes that were uniquely differentially methylated in either control- or PE-iPSC-derived trophoblast under 2% oxygen. Surprisingly, we noted a significantly higher number of DMPs in hypoxia in PE-iPSC-derived trophoblast (7668 probes uniquely hypermethylated and 5531 probes uniquely hypomethylated) compared to control-iPSC-derived trophoblast (4309 probes uniquely hypermethylated and 2680 probes uniquely hypomethylated) (Fig. 7C); this is in contrast to a smaller number of DEGs noted in hypoxia in PE-iPSC-derived trophoblast (761 unique DEGs) compared to control-iPSC-derived trophoblast (1802 unique DEGs) (see Fig. 5B). We performed functional analysis on each unique probe set using GREAT (Fig. 7C, Supplementary Figure 7), and found that probes uniquely hypomethylated in control-iPSC-derived trophoblast under hypoxia were enriched for “locomotion” (GO: 040011), “cell motility” (GO:048870), and “cell migration” (GO: 0016477) in the top 10 GO terms (Fig. 7C, Supplementary Figure 88). This was consistent with the phenotype in these cells, showing enhanced Matrigel invasion under 2% oxygen compared to invasion under 21% oxygen (see Fig. 3A). Among the genes identified through functional enrichment analysis of these DMPs, we identified genes associated with EVT differentiation and cell invasion that were uniquely enriched in control-iPSC-derived trophoblast under hypoxia. These included TWIST1 (also known as twist family bHLH transcription factor 1), and BAMBI (BMP and activin membrane bound inhibitor). TWIST1, which is a negative regulator of trophoblast cell invasion^68–70^, was uniquely downregulated 2.9-fold in control-iPSC-derived trophoblast under hypoxia. BAMBI, which mediates BMP2-induced activation of WNT/β-catenin pathway and trophoblast invasion in human primary EVT and immortalized human EVT cell lines^71^, was uniquely upregulated 3.1-fold in control-iPSC-derived trophoblast under hypoxia.

Overall, our DNA methylation analysis pointed to the presence of cell-of-origin memory, but no PE-specific marks that are retained through reprogramming into iPSC and differentiation into trophoblast, and very little genome-wide correlation with gene expression. Nevertheless, functional enrichment analysis did show some association between DMPs and DEGs with respect to the differential response of iPSCs to hypoxia, with enrichment for cell invasion-associated probes in control-iPSC-derived trophoblast under 2% oxygen.

## Discussion

Thus far, it has not been possible to model cellular defects in preeclampsia (PE), as the disease potential of early gestation CTB is unknown and the differentiation potential of CTB from third trimester tissue is limited^72^. In this study, using iPSCs reprogrammed from MSCs derived from control and PE umbilical cords, and our step-wise method of trophoblast differentiation of these cells^36^, we were able to show equivalent CTB induction, but blunted STB formation and maturation, as well as abnormal formation/function of STB and EVT in response to hypoxia, only in PE-iPSC. Both of these cellular defects have been associated with PE: specifically, spontaneous syncytialization was found to be threefold lower in CTB isolated from PE placentae^43^ and the blunted invasive capacity in response to hypoxia is consistent with previous literature suggesting a failure of placental development early in gestation, when oxygen tension is low^14^. In fact, Sheridan et al.^37^, who used a different protocol (“BAP” protocol) for trophoblast differentiation of iPSC, also noted blunted differences in invasive capacity in their PE-associated iPSC in response to changes in oxygen tension. Though these studies cannot be directly compared, due to differences in the culture conditions used for derivation, maintenance, and trophoblast differentiation of iPSC lines, the finding that two different cohorts of PE-iPSC-derived trophoblast show abnormal responses to changes in oxygen tension suggests that iPSCs can be used to recapitulate and model this complex pregnancy disorder.

In addition to the cellular phenotype, RNAseq analysis of PE-iPSC-derived trophoblast revealed similarities to differentially expressed genes (DEGs) in PE placentae. In particular, we identified multiple genes associated with protection against oxidative stress (GPX3 and GPX4)^16,44,45^, DNA damage (ATF3)^46,47^, and ER stress (RTN3)^48^, whose expression was similarly decreased in PE-iPSC-derived STB as well as in PE placentae. Since we did not identify a baseline increase in apoptosis of PE-iPSC-derived STB (data not shown), the above data suggest that this PE-associated phenotype may be secondary to the uteroplacental microenvironment, as has been previously suggested^15,73^. This indicates that in future studies, we can study not only the effect of specific stressors on these iPSC-derived STB, but also investigate strategies to protect the cells against such stress-induced apoptosis.

There were also several genes whose differential expression and function correlate with the lack of response of PE-iPSC-derived EVT to low oxygen tension, including liprin- α4 and STC1. Both of these genes promote invasion of cancer cells, and both were uniquely upregulated in hypoxia only in control-iPSC-derived EVT. Response to hypoxia is mediated primarily by hypoxia-inducible factor (HIF), which is comprised of an alpha and beta subunit^55^, of which the latter (HIF1β or ARNT) showed a modest (1.3-fold) increase only in control-iPSC-derived EVT. We have previously shown that the formation of this complex is required for differentiation of both primary and iPSC-derived CTB into EVT^10,32^; nevertheless, based on both our data and those from Sheridan et al.^37^, it appears that this subtle difference in ARNT expression under hypoxia is not associated with a defect in EVT differentiation of PE-iPSC. Rather, it is the underlying function of these cells (invasion) which is blunted under conditions (hypoxia) in which early placentation occurs^8^. In addition to changes in expression of mRNAs (as noted for liprin- α4 and STC1 above), at least part of this phenotype may be due to changes in non-coding RNAs, including MEG3 and miR-210, which regulate HIF translation and downstream signaling, respectively^58,60^. Therefore, future studies can also take advantage of the PE-iPSC model system to study mechanisms of post-transcriptional regulation of EVT invasion under different oxygen tensions.

While changes in expression of many genes in PE-iPSC-derived trophoblast, including PSG4, were consistent with the PE placental phenotype^74^, we did not see changes in genes widely known to be involved in syncytialization (such as GCM1 and SYNCYTINs): it is possible that the defect in syncytialization in these particular placentas are attributable to other, as yet unknown, genes involved in this process. In addition, we also did not see expression differences in genes coding for PE-associated circulating anti-angiogenic biomarkers (such as sFLT1 or sENG)^75,76^. The latter, however, tend to be highly variable within this patient population^77^, as also noted by Sheridan et al.^37^.

Compared to the only other iPSC-based study of PE^37^, our study went one step further, evaluating not just gene expression, but also DNA methylation changes across reprogramming and differentiation. This was done, partly because multiple studies have associated early-onset severe PE with specific patterns of DNA methylation in the placenta^78,79^, and partly because of reports indicating preservation of some cell type-specific epigenetic marks following reprogramming^62,63^. In fact, DNA methylation analysis did suggest that our iPSCs more closely resembled their cell-of-origin (umbilical cord-derived MSCs), rather than dermal fibroblasts. However, there were very few PE-specific methylation marks which were preserved through reprogramming and differentiation, and none that explained the STB formation/maturation defect.

With regard to the oxygen response phenotype, we did identify several enriched GO terms, cell motility, locomotion, and migration, among probes uniquely hypomethylated in control-iPSC-derived trophoblast, corresponding to their unique phenotype of enhanced invasion under hypoxia. However, overall, there was minimal correlation between gene expression and DNA methylation, a finding that perhaps is not surprising, given similar prior reports in human placental tissues^80^. Nevertheless, these data suggest that at least part of the “PE” phenotype resides in a molecular defect in somatic cells which manifest only during differentiation. This has been previously suggested for other iPSC-based disease models, including Parkinson’s disease^81^. Future studies should focus on assessment of chromatin state, in addition to the methylome and transcriptome, and preferably evaluate these changes, not just in iPSC, their cell-of-origin, and their trophoblast derivatives, but also in the trophoblast derived from the same placentae as the iPSCs.

It should be noted that, while our study documented this oxygen response phenotype only in iPSC-derived trophoblast, Sheridan et al.^37^ also noted such a defect in their MSCs. In fact, their PE-associated MSCs could only be cultured under low oxygen (4–5%)^39^, while ours did not show any difference in growth in either low (2%) or high (21%) oxygen, allowing us to culture and reprogram these cells into iPSC under 21% oxygen. To the extent that this phenotype is similar to the defect in invasive capacity of PE-iPSC-derived trophoblast under low oxygen, it is possible that Sheridan’s MSCs retained this phenotype following reprogramming, while ours acquired this phenotype only following trophoblast differentiation of these cells.

Our study had several limitations, however. First, compared to the study by Sheridan et al.^37^, we chose to focus on iPSCs from a smaller number of patients (3 PE and 3 non-PE), instead further defining the disease based on presence of both severe PE-specific clinical signs and symptoms and placental pathological lesions. This precluded the comparison of male and female lines; however, Sheridan et al.^37^ failed to identify any differences in their PE-iPSC phenotype based on fetal sex. In addition, we noted significant variability among both inter-patient and intra-patient iPSC clones, even in the undifferentiated state, which was perhaps not surprising, given prior studies documenting contributions of both genetic and non-genetic (i.e. chromatin) factors to clone-to-clone variability^82–85^. While this justified treating these iPSC lines independently, it also suggests that analysis of iPSC lines from additional patients is warranted in future studies. Finally, while our two-step model allowed for a more detailed analysis of trophoblast differentiation stage-specific phenotype, given the lack of lineage-specific differentiation in the second step, much of our analysis (including RNAseq and DNA methylation) was confounded due to the presence of a mixed culture of STB- and EVT-like cells^36^. In particular, as previously described^36^, EVT differentiation in these conditions was often suboptimal, accounting for at most 20–25% of the mixed culture, even under 2% oxygen. It was therefore not surprising to find only a PE-specific STB phenotype (and a greater number of PE-specific DEGs for this lineage), and not a PE-specific EVT phenotype. Our small patient cohort, driven by our strict selection criteria based on clinical and pathological presentation, did in fact show a decreased tendency toward EVT differentiation in PE-iPSCs; it is possible that with additional iPSC lines, we would have seen a statistically significant difference. In future studies, we will not only include additional iPSC lines from more patients, we also plan to take advantage of the newly-developed culture conditions for derivation and lineage-specific differentiation of human trophoblast stem cells (hTSC)^86^. We plan to adapt our iPSC-derived CTB to hTSC media, then perform STB- and EVT-specific differentiation for a more careful and detailed phenotypic and molecular study, particularly focusing on identification of PE-associated EVT-specific defects.

In conclusion, we have confirmed the utility of iPSC-based models for study of PE, and identified several cell-based phenotypic features which can be studied using this model, along with associated changes in gene expression and DNA methylation. While additional work is needed to optimize this system, this study further paves the way for modeling complex placenta-based disorders of pregnancy, allowing for study of underlying mechanisms of disease pathogenesis, but also offering a system for safe and rapid screening of therapeutics for reversing disease phenotype.

## Materials and Methods

### Generation of mesenchymal stem cells from umbilical cords

Human umbilical cord (UC) was collected aseptically under a protocol approved by the Human Research Protections Program Committee of the UCSD Institutional Review Board. (IRB number: 181917X). All patients gave informed consent for collection and use of these tissues, and all experiments were performed within guidelines and regulations set forth by the IRB. Mesenchymal stem cell (MSC) isolation was performed using a protocol modified from previous publications^38,39^. In brief, ~ 2 cm UC was washed with 2% Penicillin/streptomycin (Gemini) and 0.2% gentamicin (ThermoFisher) containing phosphate-buffered saline/PBS. UCs were minced into 1-2mm^3^ fragments using scissors and forceps, then placed at regular intervals throughout 100 mm tissue culture dishes. Once the edges of the fragments are dried and adhered (30 min to an hour) , basal media (Gibco’s alpha MEM with nucleotide, containing 10% FBS) was added in the dishes. Cultures were maintained with medium replacements every 3 days in a humidified atmosphere with 5% CO_2_ at 37 °C. After approximately 3 weeks, fibroblast-like adherent cells migrated out from the tissue fragments. The adherent cells and tissue fragments were detached using TrypLE Express (ThermoFisher), and were filtered (100 μm mesh filter; Fisherbrand) to remove tissue fragments. The cells were reseeded in T-175 flasks for further expansion in growth medium (Gibco’s alpha MEM with nucleotide, containing 10% FBS and 3 ng/ml recombinant basic fibroblast growth factor (b-FGF). All cells were positive for the mesenchymal cell marker CD73, and negative for endothelial (CD31) and leukocyte (CD45) markers, by flow cytometry. Cells were frozen down at passage 1, following negative mycoplasma testing.

### Generation of iPSC lines

MSCs (passage 3–4) were used for reprogramming in order to maximize reprogramming efficiency. MSCs were karyotyped both before and after reprogramming, to confirm normal chromosome number and confirm fetal sex. Reprogramming was done using CytoTune -iPSC 2.0 Sendai Reprogramming Kit (ThermoFisher) following the manufacturer’s protocol. In brief, reprogramming vectors were transduced into MSCs. Post-transduction day 5–7, MSCs were replated onto irraditated mouse embryonic feeder (MEF)-coated dishes with WiCell media (DMEM/F12 containing 20% Knockout serum replacement, 1X Glutamax, and 1X Nonessential amino acid, 0.1 mM 2-mercaptoethanol and 12 ng/ml b-FGF). P0 iPSC colonies appeared around 2 weeks post-transduction, and were subsequently picked for subcultures and passaged up to 10 times to dilute Sendai virus out from the culture. All the cells used in this study passed quality control assays, including confirmation of pluripotency (based on Pluritest, ThermoFisher) (Supplementary Figure 1A), normal karyotype (Karyostat, ThermoFisher) (Supplementary Figure 1B), and absence of residual expression of exogenous reprogramming factors. Cells were routinely tested for mycoplasma, and confirmed negative for all established lines.

### Human pluripotent stem cell culture and differentiation.

Trophoblast differentiation of the human pluripotent stem cells was performed under a protocol approved by the UCSD Institutional Review Board and Embryonic Stem Cell Research Oversight Committee (ESCRO Protocol number: 171648). All experiments were performed within the guidelines and regulations set by the ESCRO. Prior to differentiation, iPSCs were converted to feeder-free conditions in StemFlex (ThermoFisher) on Geltrex (ThermoFisher) coated plates (coated using 1:200 diluted Geltrex). Differentiation was performed using the two-step trophoblast differentiation established in our lab (detailed in Horii et al. 2019). iPSC cell passages 5–10 post-StemFlex adaptation were used for all experiments. XVIVO hypoxia workstation (Biospherix) was used for cell culture under 2% oxygen. Media was changed every day.

### Fusion assay

Cells grown on geltrex coated coverslips in the second step were fixed with ice cold 4% paraformaldehyde in PBS at room temperature for 10 min. Cells were then permeabilized with 0.3% Triton X-100 for 10 min and incubated with rabbit anti-ZO-1 antibody (Abcam, ab59720) as a primary antibody, and visualized by Alexa Fluor 595-conjugated goat anti-rabbit secondary antibody (ThermoFisher); nuclei were counterstained with DAPI (ThermoFisher) for 5 min. Cells and nuclei were counted in five random areas of the slide, and fusion index was calculated per the following formula: Fusion index = (number of STB nuclei – number of STB)/total number of nuclei (as previously described in Horii et al. 2016). To decrease bias, the researcher counting cells/nuclei was blinded to the study number and disease state of iPSC.

### Cell invasion assay

Transwell membranes (8.0 μm pore inserts in 24-well plate, Corning), coated with 5% Matrigel in DMEM/12, were used to perform the invasion assay. iPSC-derived CTB-like cells (over 80% EGFR^+^ by flow cytometry) were dissociated using TrypLE Express (ThermoFisher). 5 × 10^4^ cells were resuspended in 200 μl FCM with 10 ng/mL BMP4 and plated in the upper chamber of the insert, then placed in the 24 well plate containing 500 μl FCM with 10 ng/mL BMP4; media in the bottom of the chamber was changed every day. After 4 days, cells in the upper surface of the membrane were removed by a cotton swab. Cells at the bottom of the membrane were fixed with 4% PFA, and stained with mouse anti-HLA-G antibody (ab52455, Abcam), followed by Alexa Fluor 595-conjugated goat anti-mouse secondary antibody, and counterstained with DAPI (ThermoFisher). Quantification of invaded cells was done by manual counting of HLA-G^+^ cells.

### Flow cytometric analysis

Flow cytometry was conducted using live cells. Cells were collected and incubated at room temperature for one hour in FC buffer (0.5% BSA and 1% FBS in PBS) with an APC conjugated mouse anti-human EGFR antibody (clone AY13, BioLegend) and PE-conjugated mouse anti-HLA-G antibody (MEM-G/9, ExBio). APC-conjugated mouse IgG (cloneMOPC-21, BioLegend) and PE-conjugated mouse IgG (cloneMOPC-21, BioLegend) were used as isotype IgG controls. Cells were washed 3 times with FC buffer and analysis was carried out using a BD FACS-Canto Flow Cytometer.

### hCG hormone secretion assays

Cell culture supernatants were collected and stored at -80 °C until use. Levels of total hCG were quantified using the hCG ELISA Kit (HC251F, Calbiotech Inc.). The results were normalized to total DNA content, extracted by DNeasy (Qiagen), and quantified by Qubit dsDNA BR assay kit (ThermoFisher).

### RNA isolation, cDNA preparation, quantitative real-time PCR, and RNA sequencing

Total RNA was isolated using mirVana RNA Isolation Kit (Ambion). RNA concentration was measured using Qubit RNA BR assay kit (ThermoFisher). RNA integrity was checked using RNA 6000 Nano chip read by a 2100 bioanalyzer (Agilent). All samples had a RIN above 8.0.

cDNA was prepared from total RNA using the Primescipt RT-Kit (Takara bio). Quantitative real-time PCR (qPCR) was performed using TB GREEN (Takara bio) on a Quant-it Studio 5 thermocycler (ThermoFisher). The primer sequences used are TP63 (ΔNp63 isoform; F 5′-CTG GAA AAC AAT GCC CAG A-3′, R 5′-AGA GAG CAT CGA AGG TGG AG-3′), CDX2 (F 5′-TTC ACT ACA GTC GCT ACA TCA CC -3′, R 5′-TTG ATT TTC CTC TCC TTT GCT C -3′), PSG4 (F 5′-CCA GGG TAA AGC GAC CCA TT-3′, R 5′-AGA ATA TTG TGC CCG TGG GT-3′), HLA-G (F 5′- ACT GAG TGG CAA GTC CCT TT -3′, R 5′- TGG GGA AGG AAT GCA GTT CAG -3′), L19 (F 5′ -AAA ACA AGC GGA TTC TCA TGG A- 3′, R 5′ -TGC GTG CTT CCT TGG TCT TAG- 3′). Relative expression of each transcript was calculated using ΔΔC_T_ method, normalized to L19 rRNA.

RNAseq libraries were prepared using the TruSeq Stranded Total RNA Sample preparation kit with Ribo-Zero Gold (Illumina) at the IGM Genomics Center, University of California, San Diego, La Jolla, CA. Libraries were pooled and sequenced on NovaSeq 6000 S1Flow Cell (Illumina) to an average depth of 28 million uniquely mapped reads. Quality control was performed using FastQC (v. 0.11.8) and multiQC (v. 1.6), and one replicate (1938 iPS 1, day4) was eliminated from analysis (Supplementary Figure 3A,B). Reads were mapped to GRCh38.p10 (GENCODE release 26) using STAR (v. 2.7.3a)^87^ and annotated using featureCounts (subread v.1.6.3, GENCODE release 26 primary assembly annotation)^88^. The STAR parameters used were: —runMode alignReads—outSAMmode Full—outSAMattributes Standard—genomeLoad LoadAndKeep—clip3pAdapterSeq AGATCGGAAGAGC—clip3pAdapterMMp 1. The featureCounts parameters were: -s 2 -p -t exon -T 13 -g gene_id^89^. Ensembl genes without at least three samples with 10 or more reads were removed from analysis. Normalization and differential expression analysis were performed using the R (v. 3.6.3) package DEseq2 (v. 1.28.1)^90^. BiomaRt (v. 2.42.1) was used to convert Ensembl gene ID’s to HUGO gene names, and gene set enrichment analysis was done using the R(v. 3.6.3) package FGSEA (v. 1.14.0). Data visualization was done in R (v. 4.0.2) using the package gplots (v3.0.4) and Qlucore Omics Explorer (v3.6) (Qlucore).

### DNA isolation and DNA methylation analysis.

DNA was isolated using the DNeasy kit (Qiagen) and quantified using the Qubit dsDNA BR assay kit (ThermoFisher). 500 ng of DNA underwent bisulfite conversion, hybridization to the Infinium Human Methylation EPIC beadchip (Illumina) and run on an iScan (Illumina) by the IGM Genomics Center, University of California, San Diego, La Jolla, CA. Using the built-in quality control probes, all the samples passed quality control in GenomeStudio (Illumina). Preprocessing and normalization steps were done by using the R (v3.6.2) package Minfi (v1.34.0)^91^. Raw data (idat files) were combined, then normalized by noob normalization. M values were used to create PCA plots using the Qlucore Omics Explorer (v3.6) and differentially methylated probes (DMP) were detected using the dmpFinder function in Minfi (v1.34.0). Cross reacting probes^92^ and sex chromosomes were removed from the list of DMPs. Beta values were trichotomized (unmethylated, semi-methylated, and full-methylated) using a probability approach (Bayesian Gaussian Mixture model) using CpGtools (v. 1.0.2)^66^. Trichotomized probes were used for differential methylation analysis where stated. Beta values and the beta_profile_region.py script were used to create average methylation line plots using hg38.RefSeq.union.bed^66^. Functional significance of cis-regulatory regions was performed in GREAT using a 100 kb maximum extension^67^. Average methylation plots were created with CpGtools (v. 1.0.2) using the average of shown group’s median value of three samples, and the genomic region stated. Previously published samples (HDF, HDF-iPSC and H9/WA09) from Illumina’s 450 K platform^93^ were used in the data analysis. Platform-to-platform differences between the 450 k and Epic arrays were not found (Supplementary Figure 5A), nor was any difference found between PE and control MSC derived cells (Supplementary Figure 5B).

### Statistical analysis

Bar chart data display mean ± standard deviation of triplicates as stated. Box plots show median with the center line, mean as the cross mark, with the box indicating the upper and lower quartile, the whiskers indicating maximum and minimum values, and outlier/single data point being marked as circles. Statistical analysis was done using Mann-Whiney U test for independent samples, and Wilcoxon Signed Rank Test for dependent samples; * displays statistically significant values (as indicated in the figures). Differential expression analysis was performed using DEseq2 and an adjusted *p* value < 0.05 was considered differentially expressed. Differential methylation analysis was performed using Minfi, with probes with adjusted *p* value < 0.05 and Δβ-value > 0.3 considered to be differentially methylated. A delta greater than 1 was considered differentially methylated using trichotomized data.

## Supplementary Information

Supplementary Information 1.

Supplementary Information 2.

## Data Availability

RNAseq and DNA methylation data have been deposited to the Gene Expression Omnibus database under the accession number GSE159480.
