# Supplementary material for: Modeling preeclampsia using human induced pluripotent stem cells
Source: Sci Rep. 2021 Mar 15;11:5877. doi: 10.1038/s41598-021-85230-5 (PMC7961010; doi:10.1038/s41598-021-85230-5)

**Modeling Preeclampsia using human induced pluripotent stem cells**

Mariko Horii,^1,2^ Robert Morey,^2,3^ Tony Bui,^1,2^ Ojeni Touma,^1,2^ Katharine K. Nelson,^1,2^ Hee-Young Cho,^1,2,4^ Hannah Rishik,^1,2^ Louise C Laurent,^2,3^ Mana M Parast^1,2*^

^1^Department of Pathology, University of California San Diego, La Jolla, CA 92093, USA

^2^Sanford Consortium for Regenerative Medicine, University of California San Diego, La Jolla, CA 92093, USA

^3^Department of Obstetrics, Gynecology, and Reproductive Sciences, University of California San Diego, La Jolla, CA 92093, USA

^4^Department of Obstetrics and Gynecology, CHA Gangnam Medical Center, CHA University, Seoul, Korea

*****Corresponding author: Mana M. Parast, MD PhD**,** [mparast@health.ucsd.edu](mailto:mparast@health.ucsd.edu)

**Supplementary Methods**

All the methods to create supplementary data are listed in the method section in the main text.

**Supplementary Figures and Tables**

**
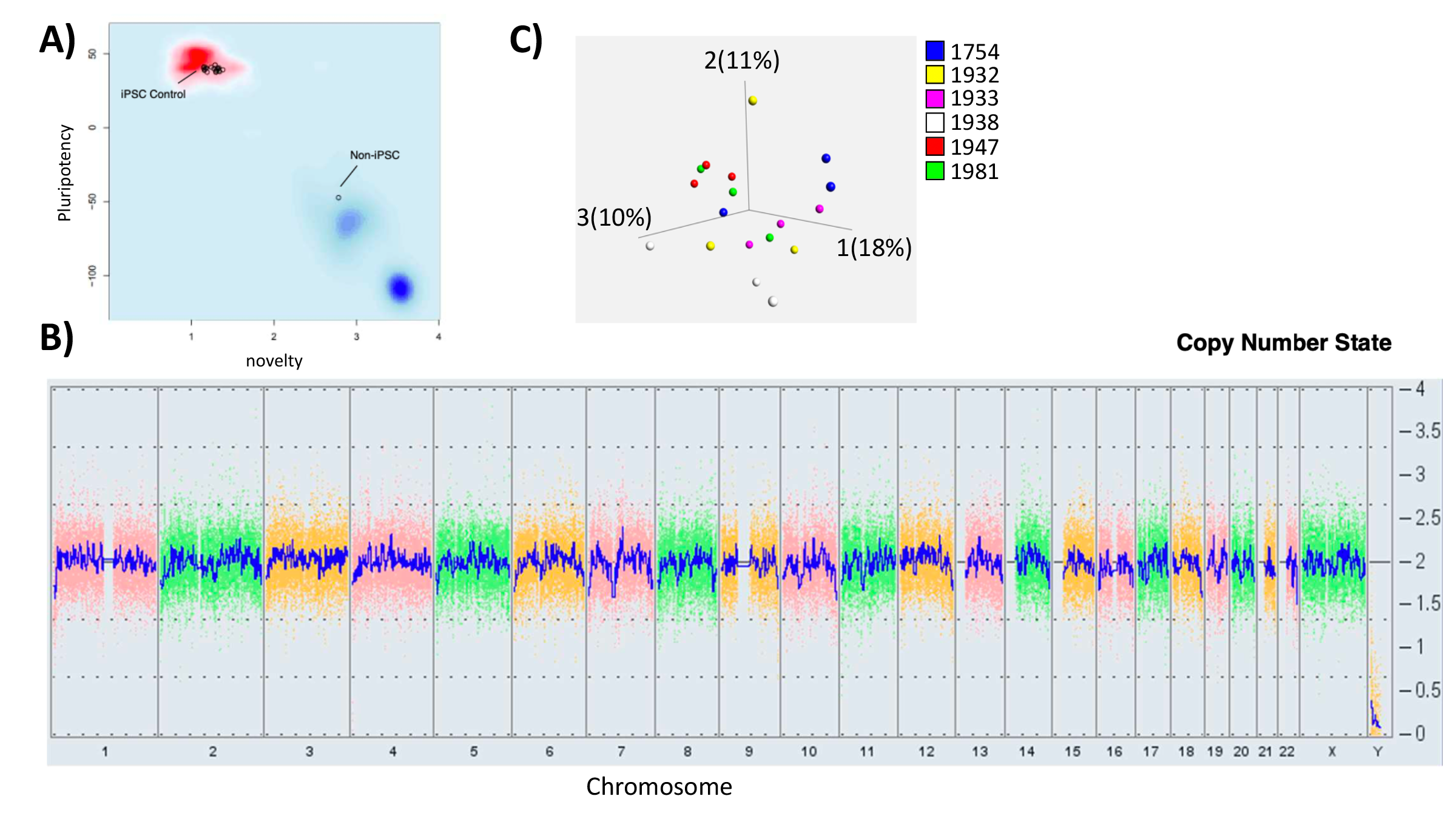
**

**Supplementary Figure 1**. **A)** Pluritest (microarray-based pluripotency assay) results of all iPSC lines (9 control and 9 PE) confirmed that all 18 iPSC lines are pluripotent (based on Pluripotency Score above 20 on the Y axis, and a Novelty Score below 1.67 on the X axis. Background displaying the empirical density distribution of all pluripotent (red cloud) and non-pluripotent/differentiated cell samples (blue clouds). **B)** Representative example of Karyostat results showing that absence of chromosomal aberrations in one iPSC line. **C)** PCA plot of RNAseq data from iPSCs showing that iPSC clones from each patient do not cluster together.

**
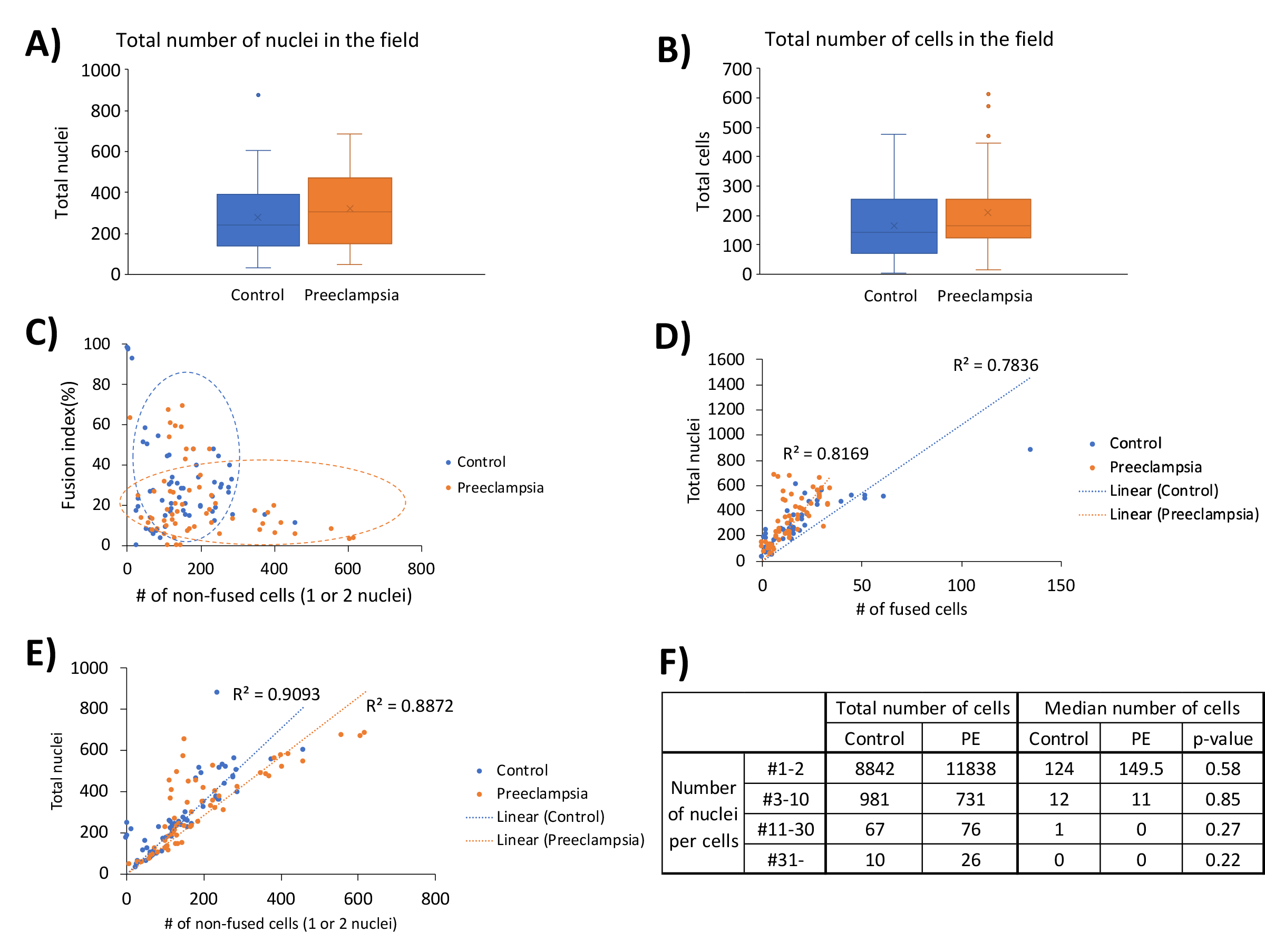
**

**Supplementary Figure 2.**

In-depth analysis of cell-cell fusion of PE and control iPSC-derived trophoblast. Box plot showing PE and control iPSC-derived trophoblast show no difference in total number of nuclei (**A**), nor in total number of cells in the field (**B**). Scatter plot of the fusion index and the number of non-fused cells **(C)** show more non-fused cells in PE-iPSC-derived trophoblast. The scatter plot of total nuclei and fused vs. non-fused cells showed fewer fused cells **(D)** and more non-fused cells **(E)**, in PE-iPSC-derived trophoblast. **(F)** Syncytialized areas of PE and control iPSC-derived trophoblast were evaluated in more detail based on the number of non-fused cells (defined as ones with 1-2 nuclei per cell), or fused cells with 3-10 nuclei, 11-30 nuclei, or 31 or more nuclei. The total number and the median number of cells per field for each category are shown. Compared to control, PE-iPSCs show more non-fused cells in the overall area; however, there was no statistically significant difference between the median cell numbers of control vs. PE in any of the categories.

**
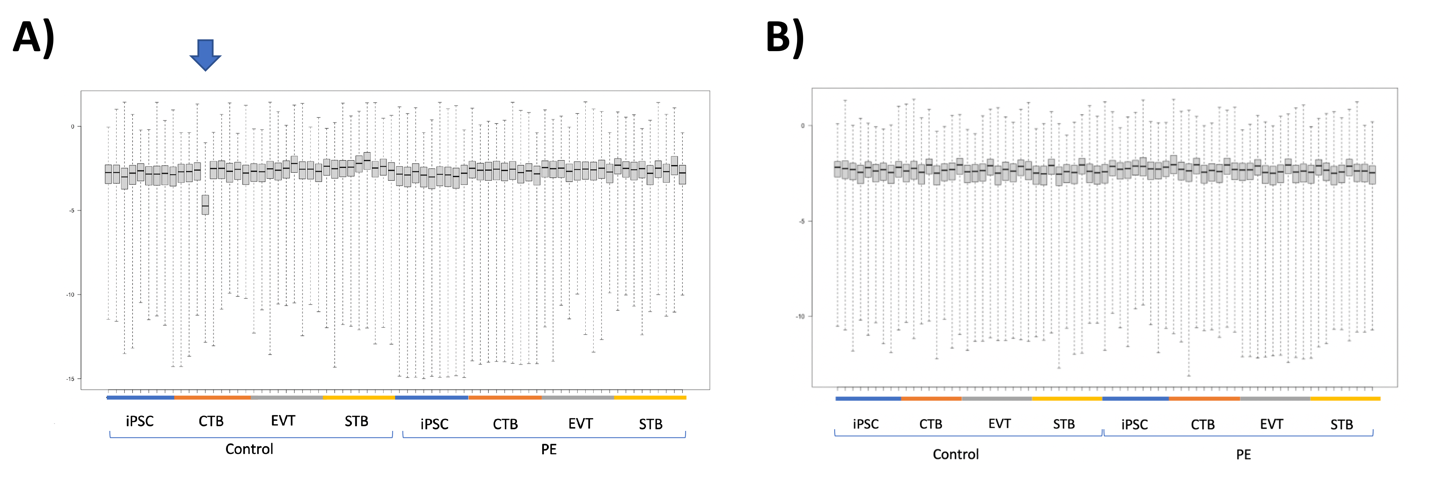
**

**Supplementary Figure 3**.

Box plot showing Cook’s distance, indicating poor sequencing data from one sample (1938 iPS1 day 4, specified by arrow) **(A)**. After removing this sample, no outliers are identified in the remaining samples in the subsequent RNAseq data analysis **(B)**.


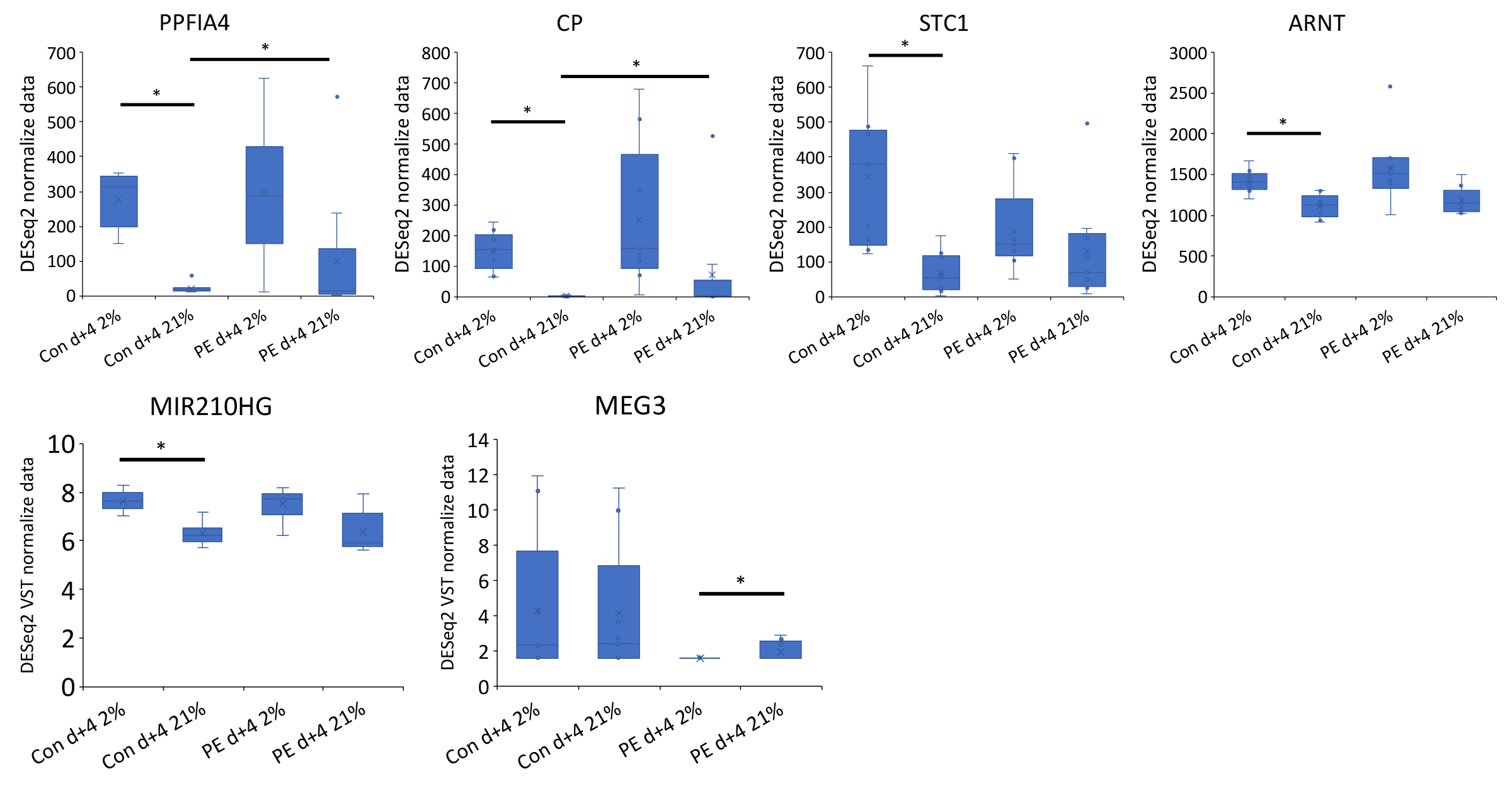


**Supplementary Figure 4**.

Box plot displaying DESeq2 normalized data of the genes highlighted in Figures 5B and 5D, plotted under each condition. *Indicates statistical significance by DESeq2 negative binomial analysis, with p-adjusted value < 0.05. (Con: Control, PE: Preeclampsia)


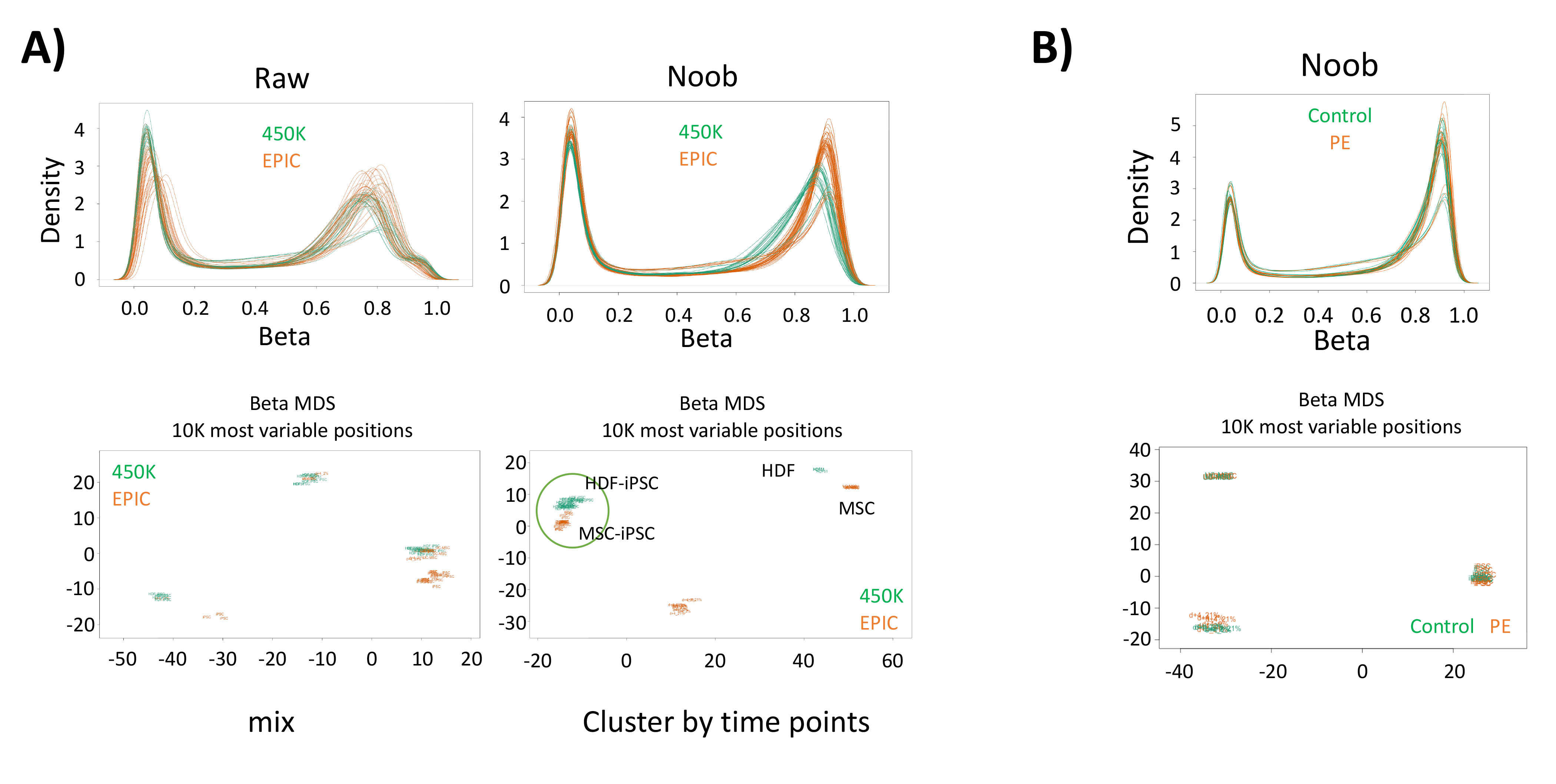


**Supplementary Figure 5**.

**A)** Density plot, and MDS plot of 10,000 most variable probes from Illumina methylation 450K (green) and EPIC (orange) array. Before normalization (Raw), the samples are variable in both density plot and MDS plot. After dye correction and normalization (Noob), density plots became cleaner, and MDS plots showed samples clustered together by their cell types but displayed no platform differences. **B)** Density plot, and MDS plot of 10,000 most variable probes from EPIC array with comparison of control (green) and PE (orange). The separation was by the time point of differentiation, but not by the disease type (PE vs. control).


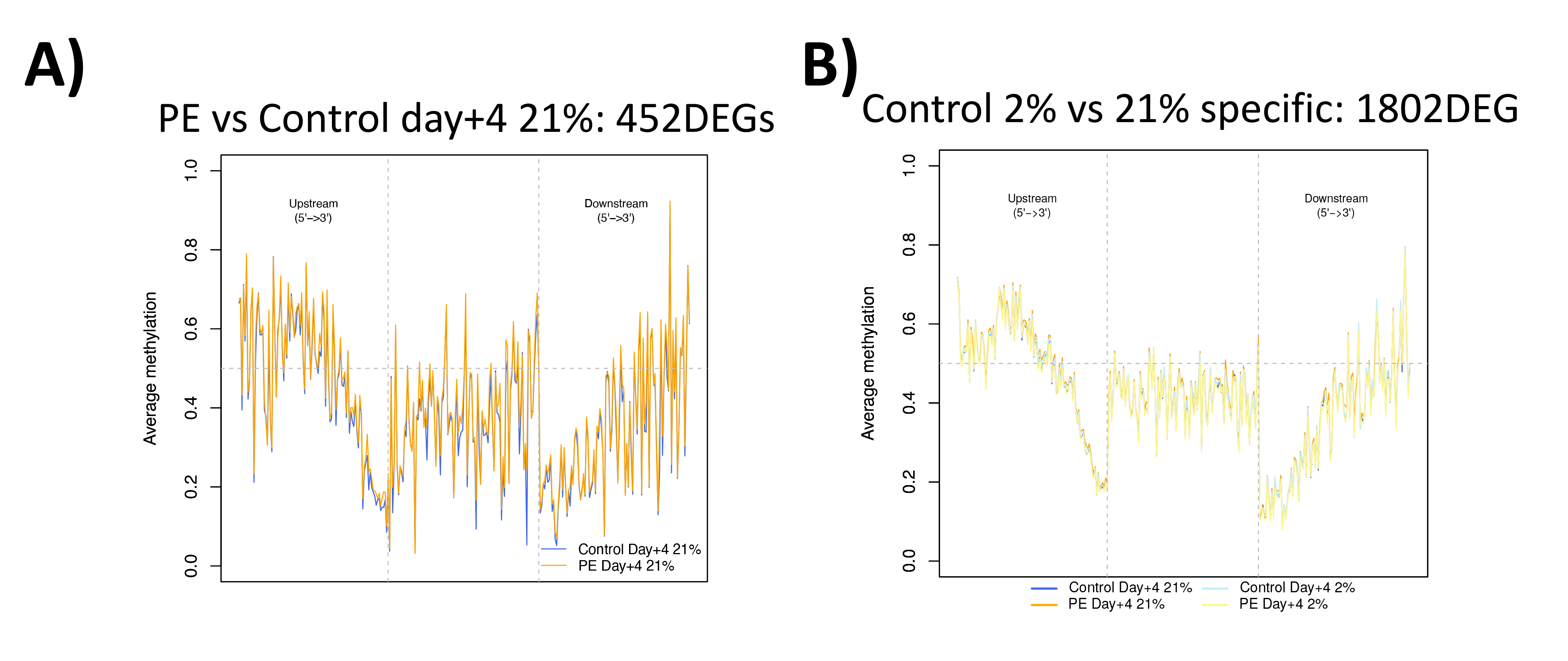


**Supplementary Figure 6**.

Average β-value within and around genes originating from **(A)** 452 DEGs, when comparing PE- vs. control-iPSC-derived trophoblast at day +4 under 21% oxygen (see **Figure 4A**), and **(B)** 1802 DEGs, uniquely enriched in control-iPSC-derived trophoblast at day +4 under 2% oxygen (see **Figure 5B**).


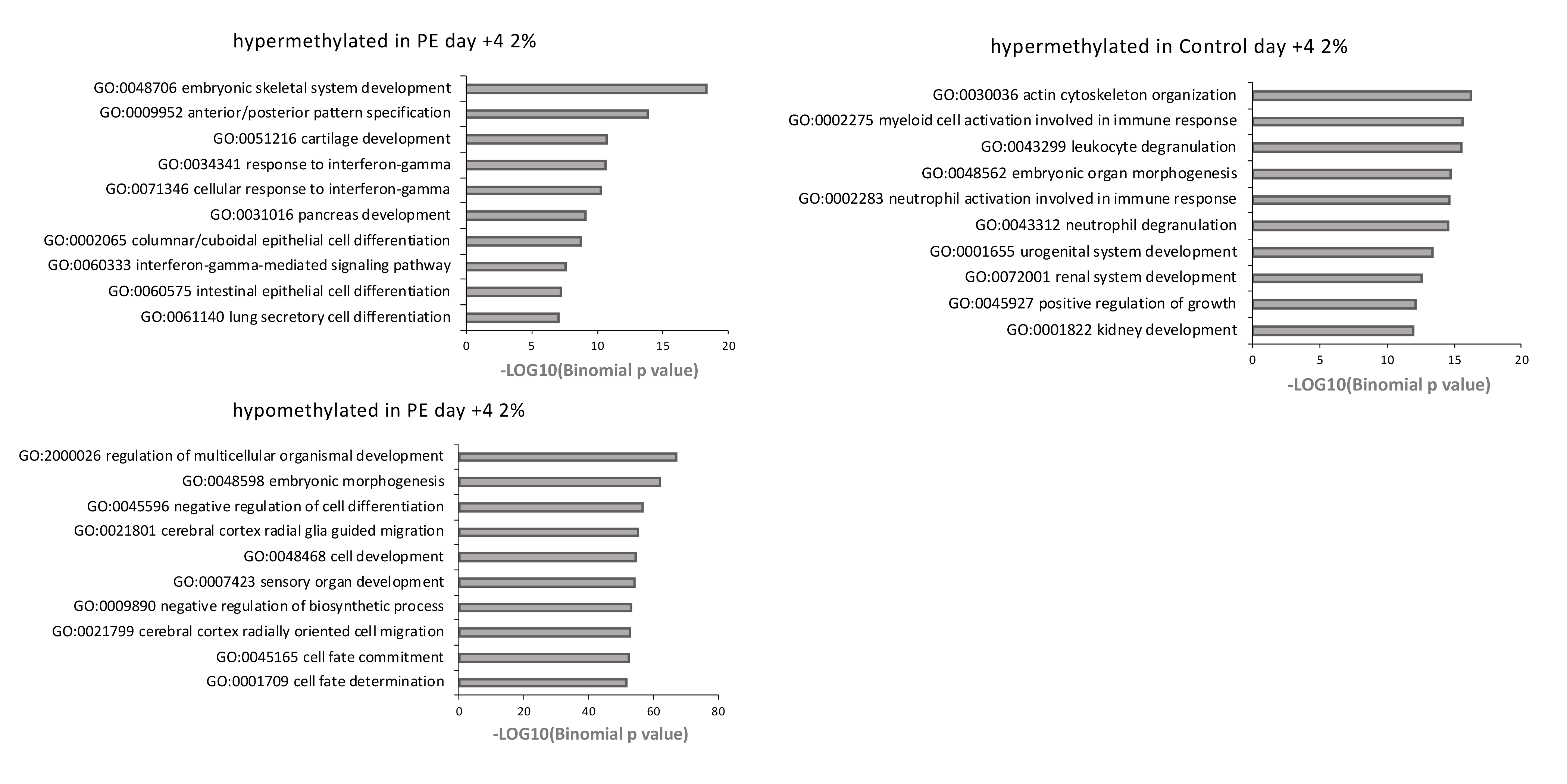


**Supplementary Figure 7**.

Top 10 GO terms from GREAT analysis summary (see **Figure 7C**), specific to DMPs from the other three categories not shown in Figure 7C (hypermethylated in PE- or control-iPSC-derived trophoblast at day +4 under 2% oxygen, and hypomethylated in PE-iPSC-derived trophoblast at day +4 under 2% oxygen.

**
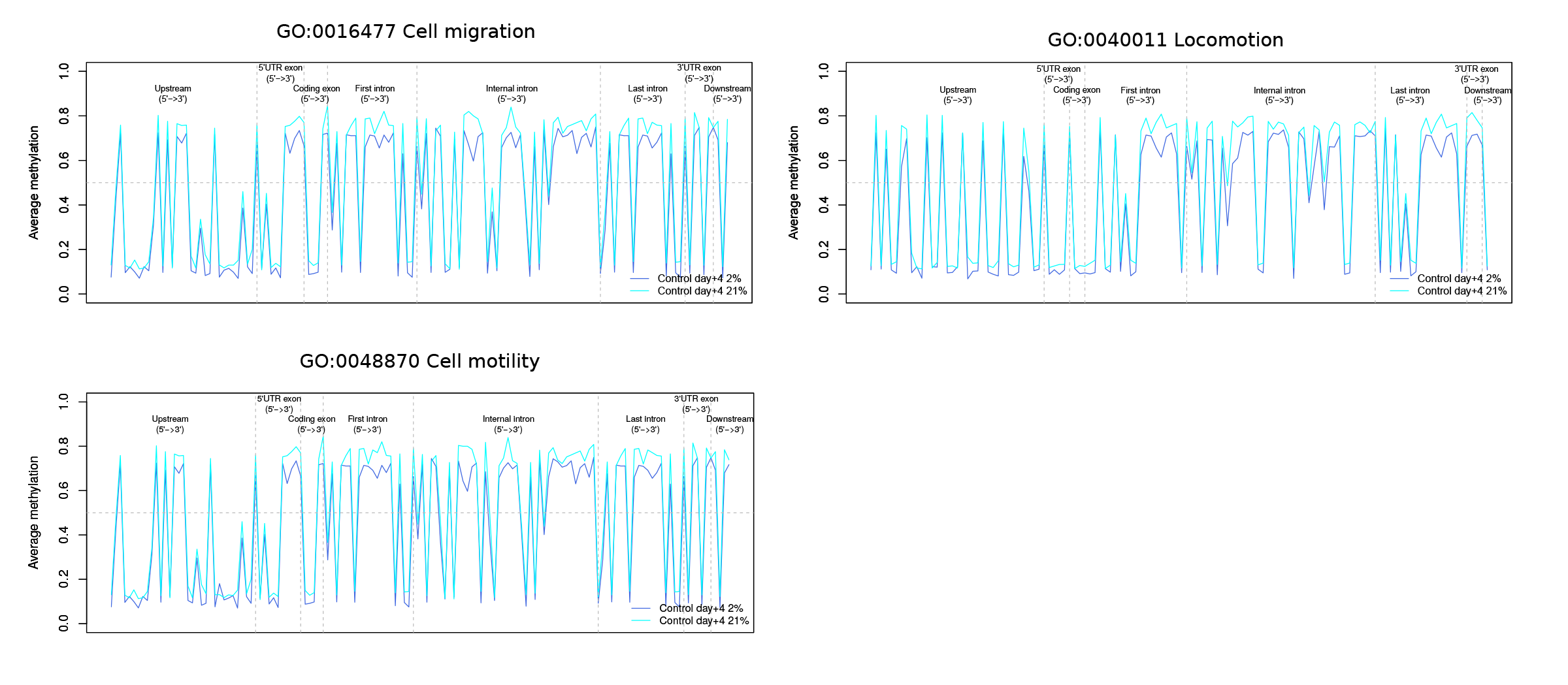
**

**Supplementary Figure 8**.

Average β-value plot of the associated probes from three highlighted GO terms in Figure 7C are plotted at each genomic region around the genes.

**Supplementary Table 1**.


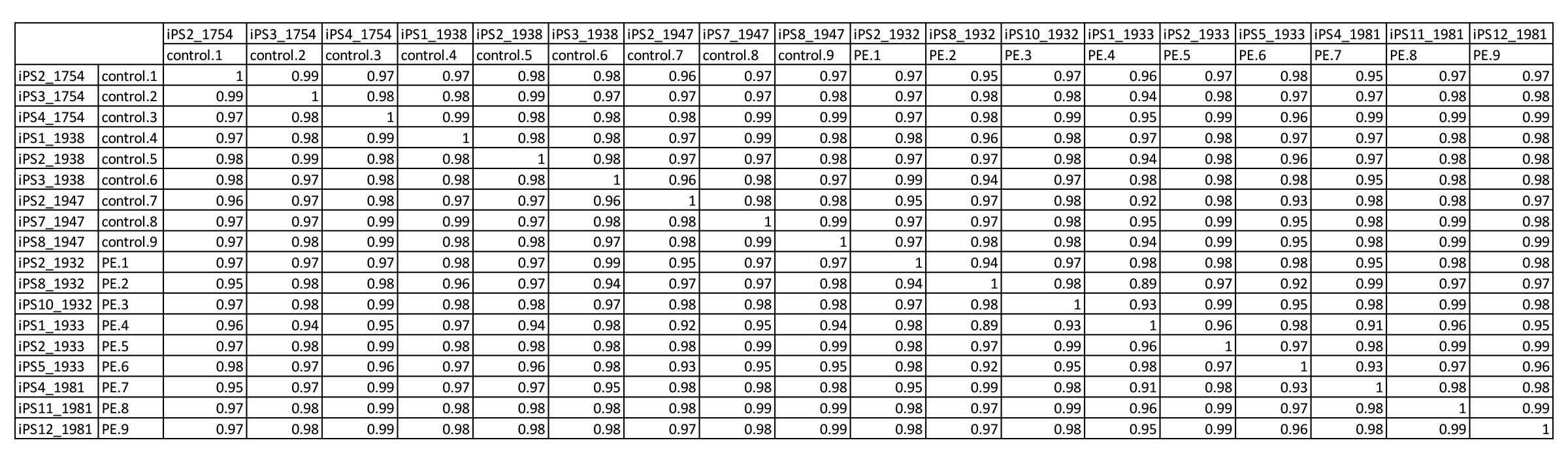
Table displaying the correlation coefficient of all the 18 iPSC lines. Calculation was done by using cor function in R using Pearson method.

**Supplementary Table 2**.

Gene Set Enrichment Analysis of PE- vs. control-iPSC-derived trophoblast at day +4 in 21% oxygen (STB-like state). Statistically enriched HALLMARK names with their normalized enrichment scores (NES), and leading edge genes (adjusted p-value < 0.05) are listed for each condition. Those in blue and orange font indicate gene-sets uniquely enriched in either control- or PE-iPSC-derived trophoblast, respectively.


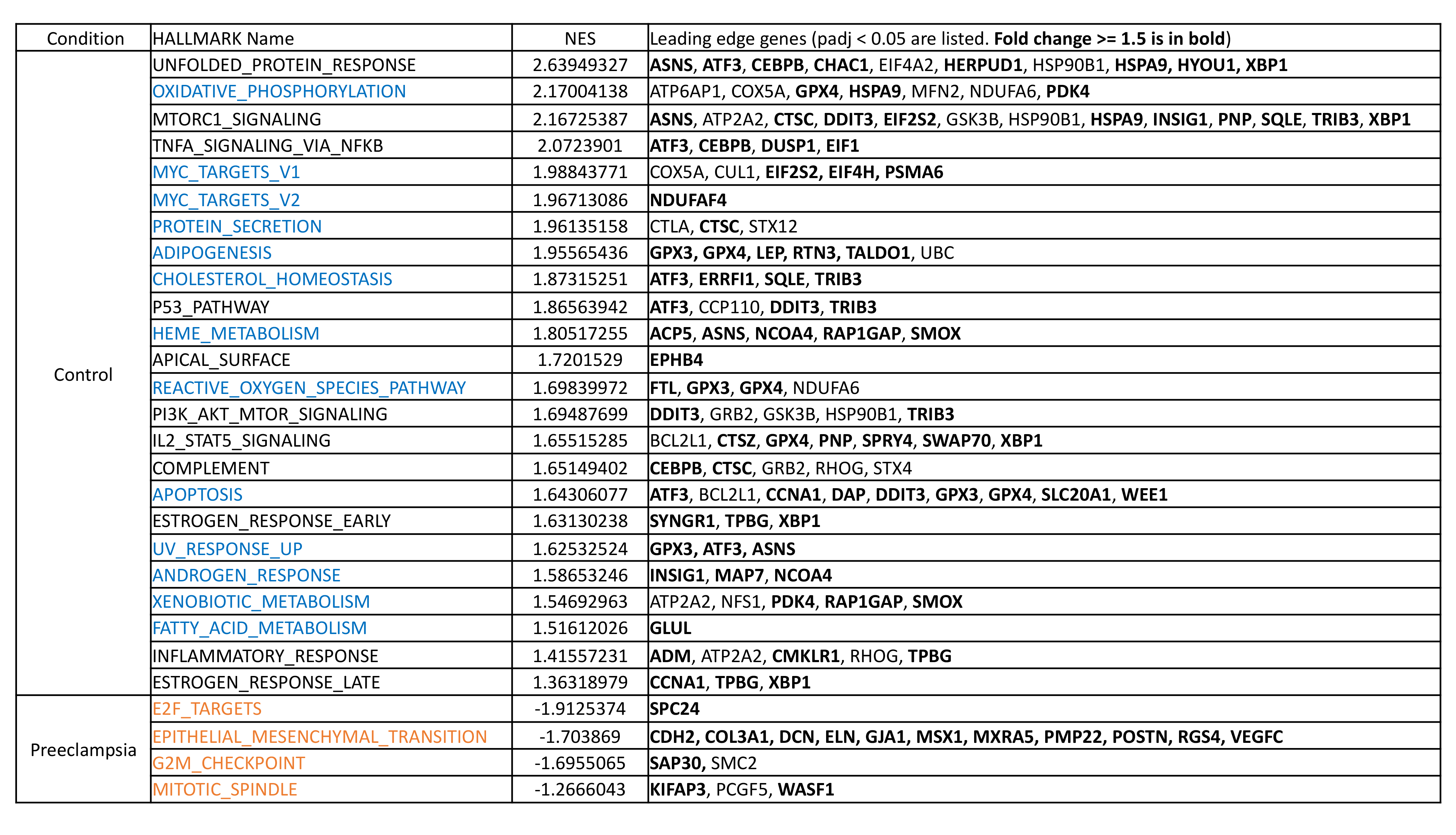


**Supplementary Table 3**.

Gene Set Enrichment Analysis data of PE- vs. control-iPSC-derived trophoblast at day +4 in 2% oxygen (EVT-like state). Only control-iPSC-derived trophoblast showed statistically significantly-enriched gene-sets; their HALLMARK names, normalized enrichment scores (NES), and leading edge genes (adjusted p-value < 0.05) are listed. The gene-set highlighted in blue is uniquely enriched in these cells.


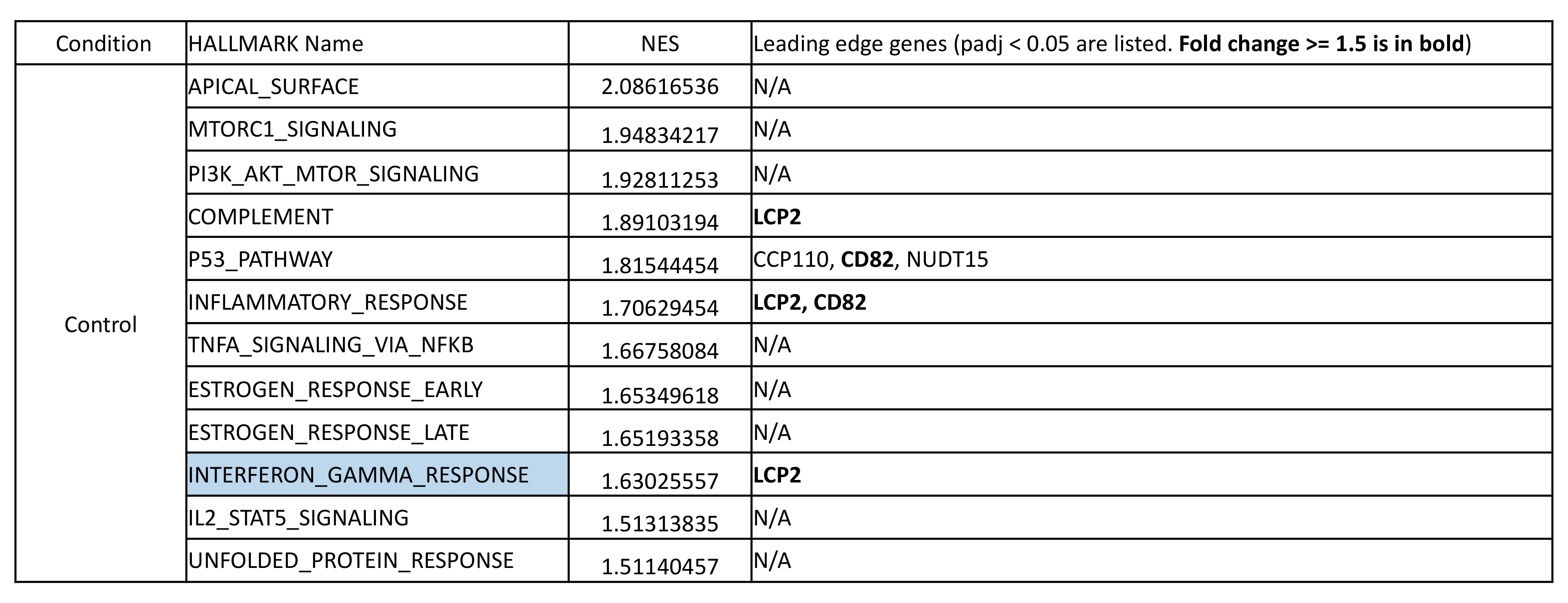

Supplement: Supplementary file 1 — Supplementary Information 1. [file 41598_2021_85230_MOESM1_ESM.docx]
